# Selenium toxicity but not deficient or super-nutritional selenium status vastly alters the transcriptome in rodents

**DOI:** 10.1186/1471-2164-12-26

**Published:** 2011-01-12

**Authors:** Anna M Raines, Roger A Sunde

**Affiliations:** 1 Department of Nutritional Sciences, University of Wisconsin, 1415 Linden Drive, Madison, WI 53706 USA

## Abstract

**Background:**

Protein and mRNA levels for several selenoproteins, such as glutathione peroxidase-1 (Gpx1), are down-regulated dramatically by selenium (Se) deficiency. These levels in rats increase sigmoidally with increasing dietary Se and reach defined plateaus at the Se requirement, making them sensitive biomarkers for Se deficiency. These levels, however, do not further increase with super-nutritional or toxic Se status, making them ineffective for detection of high Se status. Biomarkers for high Se status are needed as super-nutritional Se intakes are associated with beneficial as well as adverse health outcomes. To characterize Se regulation of the transcriptome, we conducted 3 microarray experiments in weanling mice and rats fed Se-deficient diets supplemented with up to 5 μg Se/g diet.

**Results:**

There was no effect of Se status on growth of mice fed 0 to 0.2 μg Se/g diet or rats fed 0 to 2 μg Se/g diet, but rats fed 5 μg Se/g diet showed a 23% decrease in growth and elevated plasma alanine aminotransferase activity, indicating Se toxicity. Rats fed 5 μg Se/g diet had significantly altered expression of 1193 liver transcripts, whereas mice or rats fed ≤ 2 μg Se/g diet had < 10 transcripts significantly altered relative to Se-adequate animals within an experiment. Functional analysis of genes altered by Se toxicity showed enrichment in cell movement/morphogenesis, extracellular matrix, and development/angiogenesis processes. Genes up-regulated by Se deficiency were targets of the stress response transcription factor, Nrf2. Multiple regression analysis of transcripts significantly altered by 2 μg Se/g and Se-deficient diets identified an 11-transcript biomarker panel that accounted for 99% of the variation in liver Se concentration over the full range from 0 to 5 μg Se/g diet.

**Conclusion:**

This study shows that Se toxicity (5 μg Se/g diet) in rats vastly alters the liver transcriptome whereas Se-deficiency or high but non-toxic Se intake elicits relatively few changes. This is the first evidence that a vastly expanded number of transcriptional changes itself can be a biomarker of Se toxicity, and that identified transcripts can be used to develop molecular biomarker panels that accurately predict super-nutritional and toxic Se status.

## Background

The selenoenzyme glutathione peroxidase-1 (Gpx1) is highly regulated by dietary Se, decreasing in Se deficiency to < 1% of Se adequate levels in Se-deficient liver [1]. This sensitive regulation has made Gpx1 activity a useful biomarker for determining the dietary requirement for Se, which in rodents is 0.1 μg Se/g diet (1X requirement) when based on this marker [2]. In addition to Gpx1 activity, Gpx1 mRNA is also dramatically down-regulated in Se deficiency [3], and Gpx1 mRNA can also be used to determine Se requirements [4-7]. Recently, our study on Se-regulation of the full selenoproteome in rats showed that several additional selenoprotein mRNAs are highly regulated by Se status and can also be used as biomarkers of Se deficiency [8]. The resulting Se requirements based on selenoprotein mRNAs (0.03 to 0.07 μg Se/g diet) are slightly lower than requirements based on selenoenzyme activity (0.06 to 0.13 μg Se/g diet), but importantly, none of these mRNAs are further increased in rats fed super-nutritional levels of dietary Se up to 8-times the requirement. These studies demonstrated that selenoprotein mRNAs are useful molecular biomarkers for Se deficiency, but are not effective in assessing high Se status.

Conventional biomarkers of high Se status, such as tissue Se concentration and changes in fingernail morphology, are lacking in specificity and sensitivity [9]. Molecular biomarkers are potentially better predictors of physiological effects associated with high Se intakes [10], but so far studies on the transcriptional effects of super-nutritional Se have not identified well-regulated molecular biomarkers of high Se status. In rodents several microarray studies have found 14 to 242 genes altered by a Se intake of 1.0 μg Se/g as compared to Se-deficient diets, but the only consistently regulated genes in these studies were the selenoproteins [11-13], suggesting that the Se-specific effects detected were primarily caused by Se deficiency and not high Se. Studies on the transcriptional effects of Se in various cancer cell lines and cancerous tissue have also produced variable results, with little similarity between studies [14-18]. The lack of genes consistently regulated by high Se in these microarray studies suggests that the genes so far identified may not be useful as Se-specific molecular biomarkers.

The current recommended dietary allowance (RDA) for adult humans is 55 μg/day (1X requirement), and the tolerable upper intake level (UL) for Se in humans has been set at 400 μg/day, which is about 8-times the requirement [9]. High Se intake has long been associated with prevention of cancer [19], but clinical trials of Se supplementation for cancer prevention in humans have produced inconsistent results. The Nutritional Prevention of Cancer Trial (NPCT) found that supplementation with 200 μg/day of high Se yeast (4X requirement) significantly decreased risk for prostate, lung, and colorectal cancer in patients with previous skin cancers, although no effect on recurrence of skin cancer was observed [20]. More recently, the Selenium and Vitamin E Cancer Prevention Trial (SELECT), which enrolled >35,000 men, found no effect of Se supplementation on prostate or any other cancer [21]. Follow-up studies on the NPCT subjects, however, found that Se supplementation increased the risk of squamous cell carcinoma [22], total cancer incidence [23], and diabetes [24] in subjects with higher initial plasma Se levels. Furthermore, a significant negative relationship between plasma Se level and diabetes risk was observed in National health and nutrition examination survey (NHANES) 2003-2004 subjects [25], and SELECT found a trend for increased type II diabetes risk in the Se supplemented group [21]. These reports of advantages and adverse effects associated with super-nutritional Se highlight the need to better understand the transcriptional effects of high Se intakes.

To characterize Se regulation of the transcriptome, we conducted 3 microarray studies in rodents fed diets supplemented with graded levels of Se from Se-deficient to 5 μg Se/g diet, or 50-times the requirement. Our objectives were to determine the transcriptional response to Se deficiency, super-nutritional, and toxic Se intakes in our well-characterized rodent model to identify candidates for genes regulated by high Se status, to provide insight into the molecular mechanisms underlying how animals homeostatically adapt to high Se status, and to determine whether identified Se-regulated genes could be used as molecular biomarkers of high Se status.

## Results

### Animal Growth

**Figure 1:**
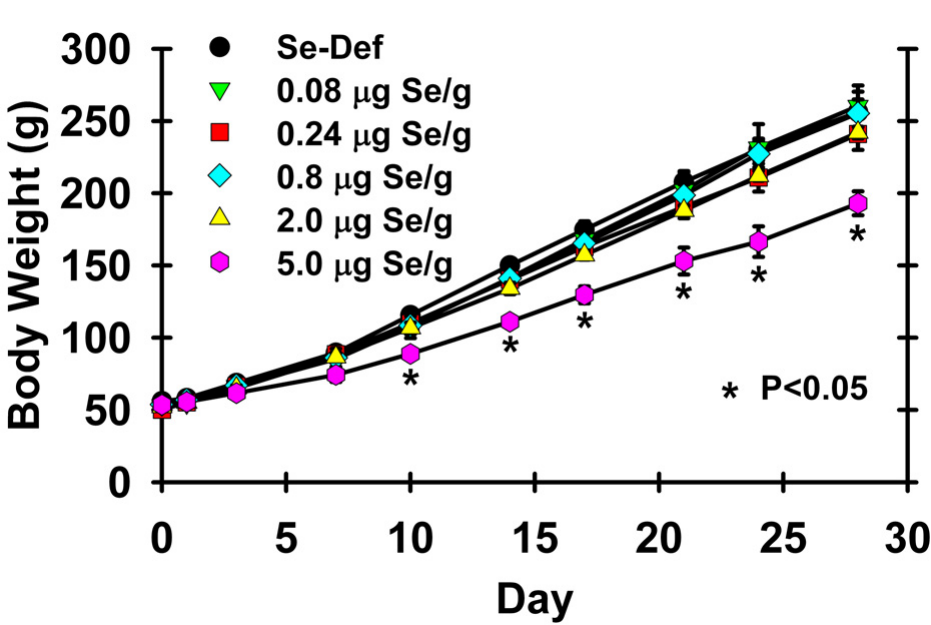
**Effect of dietary Se on rat body weight**. Male weanling rats were supplemented with 0 to 5 μg Se/g diet for 28 d and weighed bi-weekly. Values are means ± SEM, n = 4/diet. At each timepoint, values marked with an asterisk are significantly different from all other weights (P < 0.05).

In all experiments, initial body weights of treatment groups were not significantly different. In the mouse study and in rat study 1 there were no significant differences in growth due to dietary Se level [8,26], consistent with previous studies [4,5]. In rat study 2, however, rats fed 5 μg Se/g diet had significantly lower body weight as compared to all other diet groups (P < 0.05) starting at day 10 and continuing for the remainder of the study (Figure 1). In rat study 2, the initial body weight averaged 53 g, and the final average weight of rats fed 5 μg Se/g diet was 193 ± 8 g vs. an average of 251 ± 5 g (P < 0.0006) for all other diet groups.

### Se Status of Animals using Conventional Biomarkers

Se concentration in liver, Gpx1 activity in liver and red blood cells (RBCs), and Gpx3 activity in plasma were measured for each study to determine the Se status of the animals. The regulation of selenoenzyme activities in the mouse study and rat study 1 are described in detail in previous reports using these animals [8,26]. Briefly, Se concentration and Gpx1 activity in mouse liver and Gpx1 activity in RBC dropped in Se deficiency to 4, 3.5, and 37%, respectively, of Se-adequate (0.2 μg Se/g) levels, respectively. Plasma Gpx3 activity in Se-deficient mice dropped to 13% of Se-adequate levels. Liver Se and Gpx activities in mice fed the Se-marginal diet (0.05 μg Se/g diet) were intermediate between Se-deficient and Se-adequate mice. In rat study 1, liver and RBC Gpx1 activities in Se deficiency were 2 and 24%, respectively, of Se-adequate levels (0.24 μg Se/g), and increased to plateau levels by 0.09 and 0.08 μg Se/g diet, respectively. In Se-deficient rats, plasma Gpx3 activity was 2% of adequate levels and reached the plateau by 0.06 μg Se/g diet.

**Figure 2:**
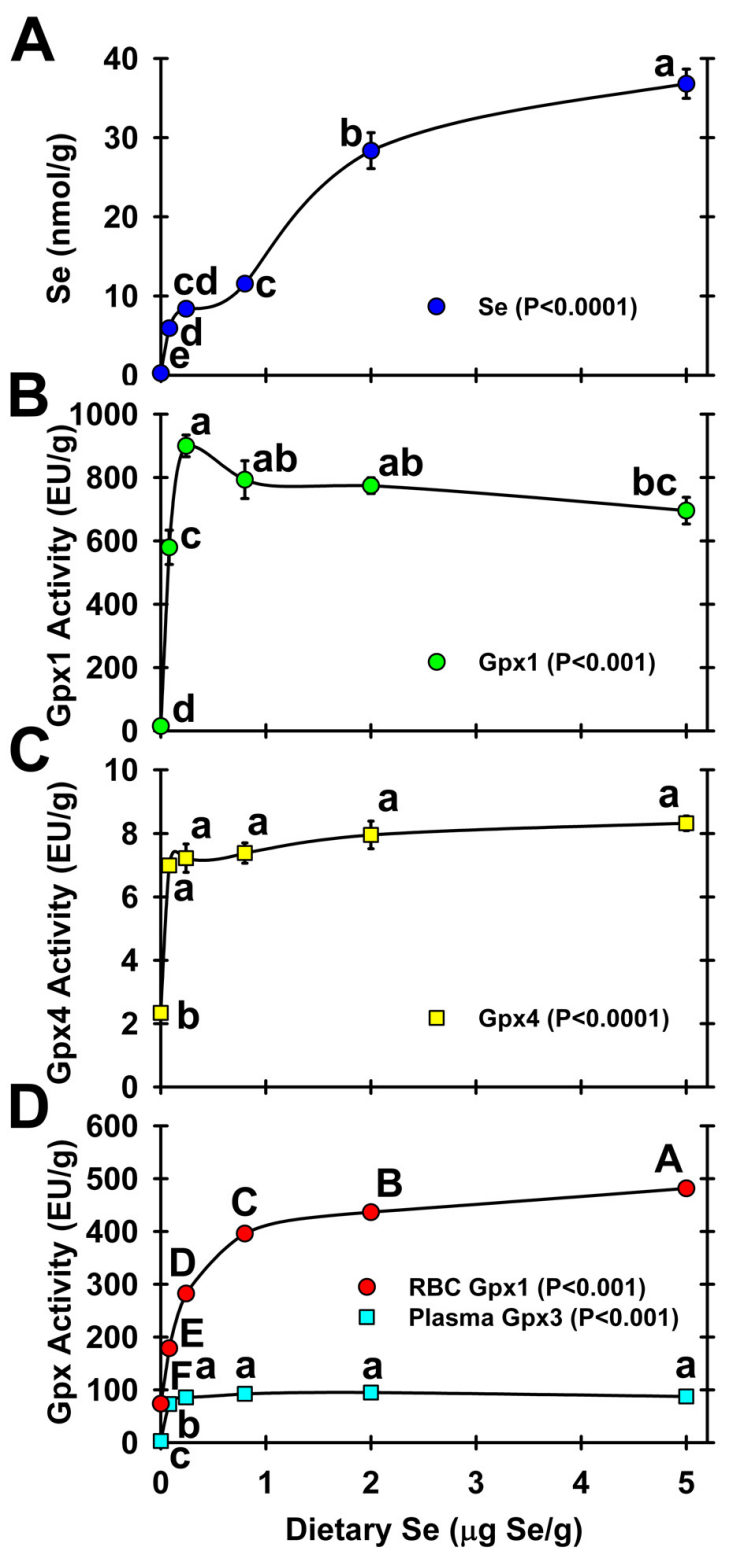
**Effect of Se status on liver Se and Gpx activities in rats**. Liver Se (A), Liver Gpx1 (B), Gpx4 (C), and RBC Gpx1 and plasma Gpx3 (D) activities in male weanling rats fed diets supplemented with 0 to 5 μg Se/g diet for 28 d. Values are means ± SEM, n = 4/diet. The level of significance by ANOVA is indicated in each panel; values with a common letter are not significantly different (P ≥ 0.05).

Liver Se concentration in Se-deficient rats in both rat study 1 and 2 decreased to 3% of Se-adequate levels (0.24 μg Se/g) (not shown and Figure 2A), similar to the mouse study. In rat study 1, liver Se increased to a plateau at 0.08 μg Se/g diet, remained at this level until 0.24 μg Se/g diet, and then increased gradually to 180% of Se adequate levels in rats fed 0.8 μg Se/g diet [8]. In rat study 2, liver Se followed exactly the same pattern as in rat study 1 (Figure 2A) and then increased to 338% and 439% of Se-adequate levels in rats fed 2 and 5 μg Se/g, respectively.

In rat study 2, liver Gpx1 activity in Se-deficient rats dropped to 2% of Se-adequate (0.24 μg Se/g) levels (Figure 2B) and liver Gpx1 activities were clearly on the plateau in rats fed >0.08 μg Se/g diet. A decrease in liver Gpx1 activity to 77% of Se-adequate levels was observed in rats fed 5 μg Se/g diet, similar to previous results [1]. This decrease may be attributed to an overall lowering of enzyme activity due to liver damage (see below). Liver Gpx4 activity in Se-deficient rats only decreased to 30% of Se-adequate levels and reached plateau levels by 0.08 μg Se/g diet (Figure 2C), as reported previously [5]. While RBC Gpx1 activity in Se-deficient rats from study 2 decreased to 26% of Se-adequate levels, RBC Gpx1 activity continued to increase with increasing levels of Se supplementation (Figure 2D) unlike the other tissues examined. This is consistent with previous reports [1,4,5], and is likely attributed to the long half life of RBCs and the relatively rapid development of the young rapidly growing rats, as RBC Gpx1 activity reaches a defined plateau in older rats [7,27]. Plasma Gpx3 activity in Se-deficient rats in rat study 2 was 3.5% of adequate, with a clear plateau above 0.08 μg Se/g diet (Figure 2C), similar to rat study 1.

### ALT and AST Activity Analyses

**Figure 3:**
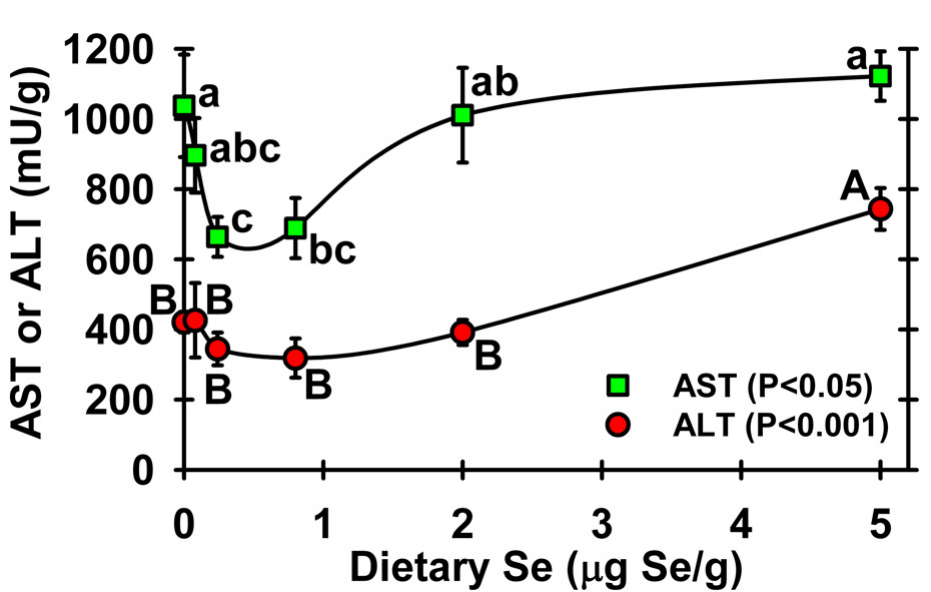
**Effect of dietary Se on markers of liver damage**. ALT and AST activities in male weanling rats fed diets supplemented with 0 to 5 μg Se/g diet for 28 d. Activities are expressed as mU/g protein. Values are means ± SEM, n = 4/diet. The level of significance by ANOVA is indicated; values with a common letter are not significantly different (P ≥ 0.05).

The reduced growth in rats fed 5 μg Se/g diet was the first indication in this study of an adverse effect of this level of dietary Se. Alanine aminotransferase (ALT) and aspartate aminotransferase (AST) activities in plasma were measured as markers of liver damage as these had previously been shown to increase at high Se intakes [28]. ALT activity was significantly increased in 5 μg Se/g diet rats as compared to all other diet groups, and was approximately twice that of Se-adequate (0.24 μg Se/g) rats (Figure 3). AST activity also showed a significant near 2-fold increase in 5 μg Se/g rats as compared to Se-adequate rats (Figure 3), but in contrast to ALT, AST activity was significantly higher in the 2 μg Se/g and Se-deficient rats as compared to Se-adequate rats, resulting in a U-shaped curve. Increased AST activity in Se-deficient plasma has been observed in other studies in our lab (unpublished results).

### Dietary Se regulation of the transcriptome

**Figure 4:**
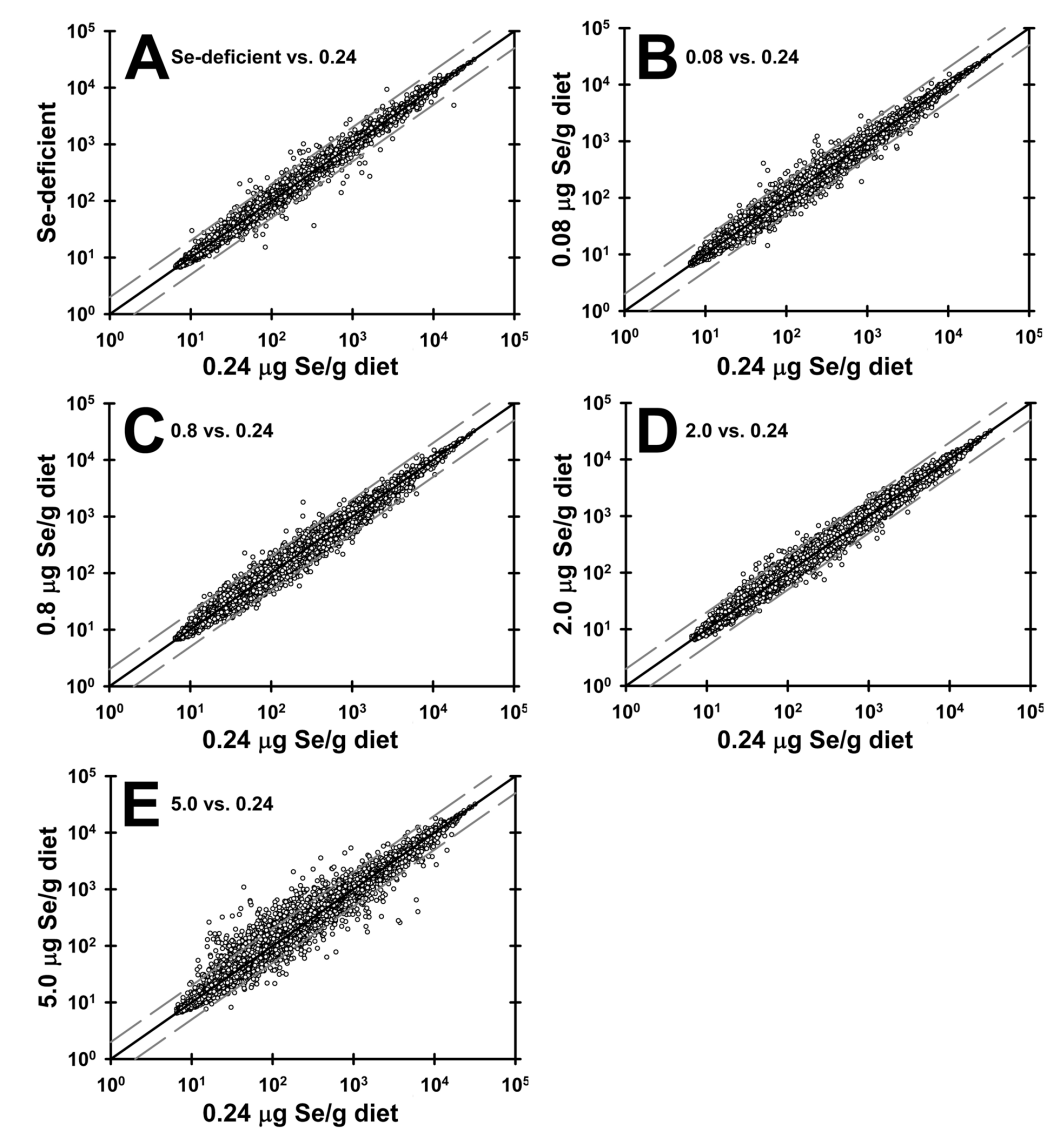
**Effect of dietary Se on the transcriptome**. RMA-generated expression values for all 31,099 probe sets on the Rat Genome 230 2.0 array were plotted for Se-deficient (A), 0.08 (B), 0.8 (C), 2 (D), and 5 μg Se/g diet (E) rat liver vs. 0.24 μg Se/g diet rat liver. Values are means, n = 3/diet. Solid black line is 1:1 expression. Dashed lines are 2-fold up- and down-regulated.

To determine the effect of Se status on general transcription, whole genome microarrays (Mouse Genome 430 2.0 and Rat Genome 230 2.0 arrays, Affymetrix, Santa Clara, CA) were used to compare gene expression in the liver or kidney of rodents fed Se-deficient and high Se diets vs. Se-adequate diets. To obtain a global picture of the effect of dietary Se on transcription, robust multichip averaging (RMA) expression for each Se treatment group (average values, n = 3/group) was plotted against expression from the Se-adequate group (0.2 μg Se/g, mouse study; 0.24 μg Se/g, rat studies). In the mouse study, this analysis showed a relatively tight clustering of expression along the 1:1 line for both 0 and 0.05 μg Se/g (data not shown). Similarly, analysis of 0.08 and 0.8 μg Se/g datasets from rat study 1 (Figure 4B, C) and of 0 and 2 μg Se/g datasets from rat study 2 showed tight 1:1 expression (Figure 4A, D). In contrast, there was an obvious expanded scattering of expression outside the 2-fold change lines in the 5 vs. 0.24 μg Se/g diet analysis (Figure 4E), clearly showing the unique transcriptional effects elicited by toxic Se intake. The tightness of expression in the 0.8 vs. 0.24 μg Se and 2 vs. 0.24 μg Se analyses are particular striking, given that these levels are 8 and 20 times the Se requirement in the rat [2,4].

### Transcriptional regulation by Se deficiency

Se-deficient treatment groups were included in these microarray studies in order to assess Se status across the full range from Se-deficient to toxic Se status, to further characterize the effect of Se deficiency on the transcriptome, and to be able to distinguish the effects of Se deficiency from Se excess. Our previous studies [8,26] showed that expression of a subset of the 24 rodent selenoproteins is down-regulated by Se deficiency. For these studies the full complement of non-selenoprotein genes was evaluated for regulation by Se status. In the mouse study, Se deficiency significantly up-regulated 3 genes in liver (Cbr3, Abcc4, and Hspb1), with 4 additional genes nearly significant (P < 0.2) as compared to Se-adequate (0.2 μg Se/g) mice (Table 1). No genes were significantly up-regulated in Se-deficient kidney as compared to Se-adequate kidney (Table 1). Similarly, in rat study 2 there were only 2 genes significantly up-regulated (Gsta5 and LOC286989) in Se-deficient vs. Se-adequate (0.24 μg Se/g) rats (Table 1). A few genes or gene families are up-regulated in all 3 Se-deficient datasets, although these did not reach significance in every treatment. NAD(P)H dehydrogenase, quinone 1 (Nqo1), and members of the ATP-binding cassette subfamily C and glutathione transferase-alpha family were consistently up-regulated by Se deficiency.

**Table 1:** Transcripts up-regulated by Se deficiency

Genbank Accession	Gene Symbol	Gene Title	Fold Change^1^	adj P-value^2^	Nrf2 Target^3^
**Mouse Liver**					
NM_173047	Cbr3	carbonyl reductase 3	7.46	1.7E-05	X
NM_001033336	Abcc4	ATP-binding cassette, sub-family C (CFTR/MRP), member 4	3.73	0.023	X
NM_013560	Hspb1	heat shock protein 1	1.91	0.04	
			- - - - - - - - - - - - - - - - - - - - - - - - -		
NM_008182	Gsta2	glutathione S-transferase, alpha 2	1.93	0.057	
NM_007620	Cbr1	carbonyl reductase 1	1.89	0.057	X
NM_008706	Nqo1	NADPH dehydrogenase, quinone 1	1.85	0.073	X
NM_010357	Gsta4	glutathione S-transferase, alpha 4	1.74	0.17	X
**Mouse Kidney**					
			- - - - - - - - - - - - - - - - - - - - - - - - -		
NM_001077353	Gsta3	glutathione S-transferase, alpha 3	1.74	0.076	X
NM_001033336	Abcc4	ATP-binding cassette, sub-family C (CFTR/MRP), member 4	1.89	0.11	X
NM_008706	Nqo1	NADPH dehydrogenase, quinone 1	1.64	0.29	X
**Rat Liver^4^**					
NM_173323	Ugt2b7	UDP glucuronosyltransferase 2 family, polypeptide B7	2.14	0.01	
NM_001009920	Gsta5	glutathione S-transferase Yc2 subunit	3.03	0.02	
			- - - - - - - - - - - - - - - - - - - - - - - - -		
NM_080581	Abcc3	ATP-binding cassette, sub-family C (CFTR/MRP), member 3	4.29	0.07	X
NM_033443	Arsb	arylsulfatase B	1.40	0.07	
NM_017000	Nqo1	NADPH dehydrogenase, quinone 1	1.99	0.16	X

Transcripts that were significantly (above dashed line, P < 0.05) or nearly significantly (below dashed line, 0.05 < P < 0.2) up-regulated in Se-deficient rodent tissues compared to Se-adequate (0.2 or 0.24 μg Se/g diet, for mouse and rat studies respectively). ^1^Fold changes determined by analysis of RMA expression data with the Limma package in R software, ^2^P-values were adjusted for multiple testing, ^3^Genes known to be Nrf2 targets, determined as described in the text, ^4 ^Genes up-regulated in Se-deficient liver from rats in study 2.

Se deficiency significantly down-regulated 5 genes in mouse liver, 3 genes in mouse kidney, and 4 genes in rat liver; these were all selenoproteins previously reported to be highly regulated by Se [8,26] (Table 2), and there were no additional non-selenoprotein transcripts down-regulated by Se deficiency. qRT-PCR confirmed microarray-detected Se-regulation for selected genes up-regulated in Se deficiency, found expression levels up to 8-fold higher in Se-deficient mouse liver, and showed that these genes were restored to adequate levels in the mice fed a Se marginal diet (0.05 μg Se/g diet) (Additional file 1, Figures S1A-S1F). In rats, qRT-PCR confirmed that Abcc3 and Nqo1 were increased by Se deficiency 4-fold and 2-fold relative to Se-adequate levels, but also found that Abcc3 was increased by 5 μg Se/g diet to levels not different from levels in Se-deficient rat liver (Additional file 1, Figures S1G, S1H).

**Table 2:** Transcripts down regulated by Se deficiency

Genbank Accession	Gene Symbol	Gene Title	Fold Change^1^	adj P-value^2^	Nrf2 Target^3^
**Mouse Liver**					
NM_009156	Sepw1	selenoprotein W, muscle 1	-3.73	1.7E-05	
NM_001033166	Selh	selenoprotein H	-2.83	0.0009	
NM_008160	Gpx1	glutathione peroxidase 1	-2.14	0.0001	
NM_019979	Selk	selenoprotein K	-2.14	0.0012	
NM_013711	Txnrd2	thioredoxin reductase 2	-1.64	0.0091	
			- - - - - - - - - - - - - - - - - - - - - - - - -		
NM_015762	Txnrd1	thioredoxin reductase 1	-1.57	0.099	X
**Mouse Kidney**					
NM_001033166	Selh	selenoprotein H	-2.64	0.0004	
NM_009156	Sepw1	selenoprotein W, muscle 1	-2.64	0.0044	
NM_008160	Gpx1	glutathione peroxidase 1	-2.46	0.0039	
			- - - - - - - - - - - - - - - - - - - - - - - - -		
**Rat Liver^4^**					
NM_001114939	Selh	selenoprotein H	-6.06	< 0.001	
NM_030826	Gpx1	glutathione peroxidase 1	-3.73	< 0.001	
NM_001014253	Selt	selenoprotein T	-1.60	0.005	
NM_001106609	Txnrd3	thioredoxin reductase 3	-1.60	0.03	
			- - - - - - - - - - - - - - - - - - - - - - - - -		
NM_022584	Txnrd2	thioredoxin reductase 2	-1.57	0.06	
NM_207589	Selk	selenoprotein K	-2.46	0.07	

Transcripts that were significantly (above dashed line, P < 0.05) or nearly significantly (below dashed line, 0.05 < P < 0.2) down-regulated in Se-deficient rodent tissues compared to Se-adequate (0.2 or 0.24 μg Se/g diet, for mouse and rat studies respectively). ^1^Fold changes determined by analysis of RMA expression data with the Limma package in R software, ^2^P-values were adjusted for multiple testing, ^3^Genes known to be Nrf2 targets, determined as described in the text, ^4^Genes down-regulated in Se-deficient liver from rats in study 2.

### Transcriptional Effects of High Se

To determine whether there are transcripts regulated by super-nutritional and toxic Se intakes, microarrays were used to analyze expression in rats fed 8, 20 and 50-times the Se requirement as compared to Se-adequate rats. Rat study 1 determined the effect of Se status in rats fed up to 0.8 μg Se/g, 8-times the requirement as compared with Se-adequate rats (0.08 μg Se/g diet). Surprisingly, there were no significant gene expression changes with this 10-fold increase in Se intake. When compared to Se-adequate rats from study 2 (0.24 μg Se/g diet), there were 84 significant gene changes in rats fed 0.8 μg Se/g diet, but these changes are likely due to differences between these independent experiments rather than due to Se status. RMA expression data plotted for several of the transcripts altered in the 0.8 vs. 0.24 μg Se/g diet comparison show that expression is changed at 0.8 and to a lesser extent at 0.08, while remaining constant at the other Se intakes (data not shown).

As no significant gene expression changes were observed at 8-times the requirement, a second rat study was conducted to assess transcriptional effects of Se intakes at 20 and 50-times the requirement (2 and 5 μg Se/g diet). Gene expression in rats fed 2 and 5 μg Se/g diet was compared with Se adequate rats (0.24 μg Se/g diet). Only 6 transcripts (5 genes and 1 EST) were significantly changed by 2 μg Se/g, with 5 others nearly significant (P < 0.2) (Table 3). The genes up-regulated at 2.0 μg Se/g diet were: Rgs4, a regulator of g-protein signaling; Ccdc80, which may be involved in extracellular matrix organization; RGD1560666, a gene of unknown function that contains putative signaling domains; and Oxct1, a member of the 3-oxoacid CoA-transferase family. The lone down-regulated gene at 20-times the requirement was Cirbp, which contains RNA binding domains and is thought to stabilize mRNAs and enhance translation.

**Table 3:** Transcripts regulated by 2 μg Se/g diet

Genbank Accession	Gene Symbol	Gene Title	Fold Change^1^	adj P-value^2^	Nrf2 Target^3^
NM_017214	Rgs4	regulator of G-protein signaling 4	3.03	0.005	X
NM_022543	Ccdc80	coiled-coil domain containing 80	2.14	0.02	
XM_001069576	RGD1560666	Similar to KIAA1280 protein	1.80	0.02	
NM_001127580	Oxct1	3-oxoacid CoA transferase 1	1.64	0.02	
NM_031147	Cirbp	cold inducible RNA binding protein	-1.83	0.02	
BE107169	EST	UI-R-BS1-ayq-f-06-0-UI.s1	1.70	0.03	
			- - - - - - - - - - - - - - - - - - - - - - - - -		
NM_001009965	Tsku	Tsukushin	2.30	0.13	X
NM_001011901	Hsph1	heat shock 105 kDa/110 kDa protein 1	1.68	0.13	
NM_012886	Timp3	TIMP metallopeptidase inhibitor 3	1.68	0.13	
AA945268	EST	EST200767	-3.25	0.19	
NM_001164396	RGD1564865	similar to 20-alpha-hydroxysteroid dehydrogenase	-2.14	0.13	

Transcripts that were significantly (above the dashed line, P < 0.05) or nearly significantly (below the dashed line, 0.05 < P < 0.2) differentially expressed in liver from rats fed 2 μg Se/g diet compared to 0.24 μg Se/g diet. ^1^Fold changes determined by analysis of RMA expression data with the Limma package in R software, ^2^P-values were adjusted for multiple testing, ^3^Genes known to be Nrf2 targets, determined as described in the text.

**Figure 5:**
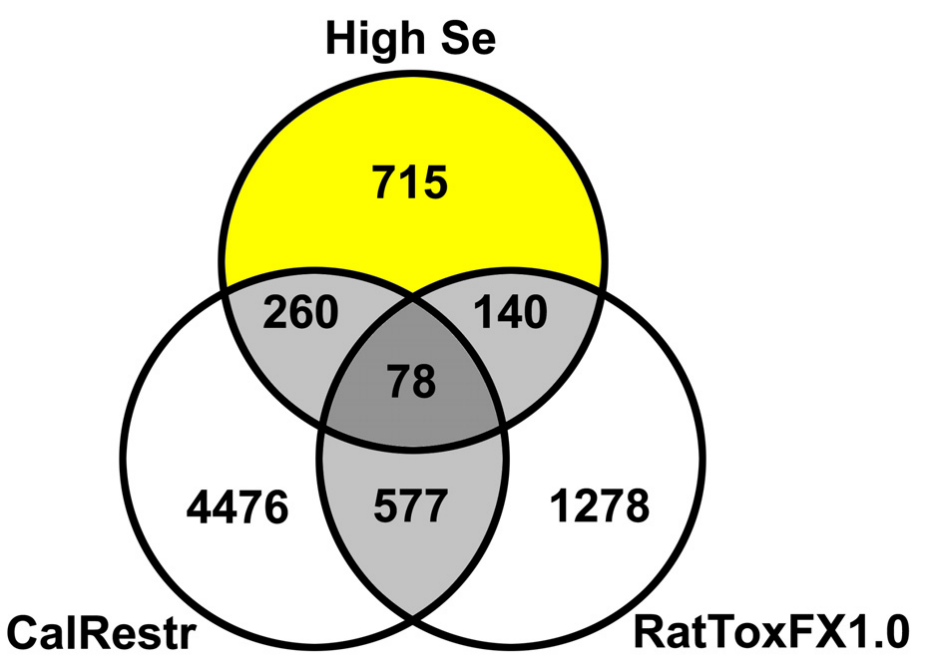
**Identification of Se-specific probe sets regulated by 5 μg Se/g diet**. The 1193 probe sets that were changed significantly by 5 μg Se/g diet in rat study 2 as compared to 0.24 μg Se/g diet were filtered to remove 260 non-specific transcripts overlapping with probe sets changed significantly by caloric restriction (CalRestr), to remove 140 transcripts included in the RatTox FX 1.0 array, and to remove 78 transcripts found in both datasets. The resulting 715 high Se-transcript dataset contains 48 duplicate transcripts, yielding 667 unique transcripts which correspond to 437 unique genes.

In contrast to the few transcripts changed at 20-times the Se requirement, there were 1193 transcripts significantly altered at 50-times the requirement. As the rats fed 5 μg Se/g diet had reduced growth and elevated markers of liver damage, it was possible that many of these transcripts could be responding to general toxicity and/or caloric restriction. Therefore, the 1193 Se toxicity transcripts that overlapped with Affymetrix's RatToxFX 1.0 array (218 of 2073 probe sets) and that overlapped with 10 day caloric restriction (CalRestr) data from a recent study (338 of 5391 probe sets) [29] were removed, along with redundant transcripts for genes found in RatTox or CalRestr datasets (Figure 5). After filtering, 715 Se-specific transcripts remained in this Se-specific dataset. This 715-transcript dataset contained 48 duplicate transcripts, yielding 667 unique transcripts which correspond to 437 unique genes for subsequent GOMiner analyses. Most of the unique transcripts were up-regulated, with 542 up- vs. 125 down-regulated. GOMiner analysis identified 33 biological processes significantly enriched in the Se-specific dataset, and these were nearly all related to cell movement/morphogenesis, extracellular matrix (ECM), and development/angiogenesis (Table 4).

**Table 4:** Biological Processes enriched in Se-specific genes

GO Identifier	Term	Total Genes^1^	Changed Genes^2^	Enrich ment	FDR^c^
**Cell movement/morphogenesis**					
GO:0006929	substrate-bound_cell_migration	11	4	9.37	0.032
GO:0007155	cell_adhesion	586	47	2.07	0.000
GO:0022610	biological_adhesion	586	47	2.07	0.000
GO:0008360	regulation_of_cell_shape	49	11	5.78	0.000
GO:0022604	regulation_of_cell_morphogenesis	126	18	3.68	0.000
GO:0000902	cell_morphogenesis	386	29	1.94	0.021
GO:0032989	cellular_component_morphogenesis	423	34	2.07	0.000
GO:0051128	regulation_of_cellular_component_organization	429	32	1.92	0.014
**Extra cellular matrix**					
GO:0030199	collagen_fibril_organization	23	6	6.72	0.006
GO:0030198	extracellular_matrix_organization	88	14	4.10	0.000
GO:0030036	actin_cytoskeleton_organization	248	25	2.60	0.000
GO:0043062	extracellular_structure_organization	156	15	2.48	0.045
GO:0030029	actin_filament-based_process	263	25	2.45	0.000
GO:0007010	cytoskeleton_organization	417	33	2.04	0.001
**Development/Angiogenesis**					
GO:0010926	anatomical_structure_formation	980	59	1.55	0.018
GO:0048856	anatomical_structure_development	2224	132	1.53	0.000
GO:0009653	anatomical_structure_morphogenesis	1158	78	1.74	0.000
GO:0022603	regulation_of_anatomical_structure_morphogenesis	260	27	2.68	0.000
GO:0048646	anatomical_structure_formation_involved_in_morphogenesis	346	30	2.23	0.000
GO:0001570	Vasculogenesis	40	8	5.15	0.004
GO:0001525	Angiogenesis	182	19	2.69	0.002
GO:0001568	blood_vessel_development	278	27	2.50	0.000
GO:0048514	blood_vessel_morphogenesis	237	23	2.50	0.000
GO:0001944	vasculature_development	282	27	2.47	0.000
GO:0009887	organ_morphogenesis	735	47	1.65	0.021
GO:0048731	system_development	2107	119	1.46	0.000
GO:0048869	cellular_developmental_process	1545	87	1.45	0.003
GO:0048513	organ_development	1633	91	1.44	0.004
GO:0007275	multicellular_organismal_development	2439	130	1.37	0.000
GO:0032502	developmental_process	2955	148	1.29	0.003
**Other**					
GO:0034329	cell_junction_assembly	41	7	4.40	0.043
GO:0007165	signal_transduction	2543	127	1.29	0.020
GO:0032501	multicellular_organismal_process	3222	154	1.23	0.039

Gene ontology (GO) identifiers significantly enriched in the 667 Se-specific genes. ^1^Number of genes involved in this process in the set of all genes on the Rat Genome 230 2.0 Array, ^2^Number of genes involved in this process in the set of genes specific to Se toxicity, ^3^False Discovery Rate - ratio of the number of times the Se toxicity data gave P < 0.05 compared to a random set of data

**Figure 6:**
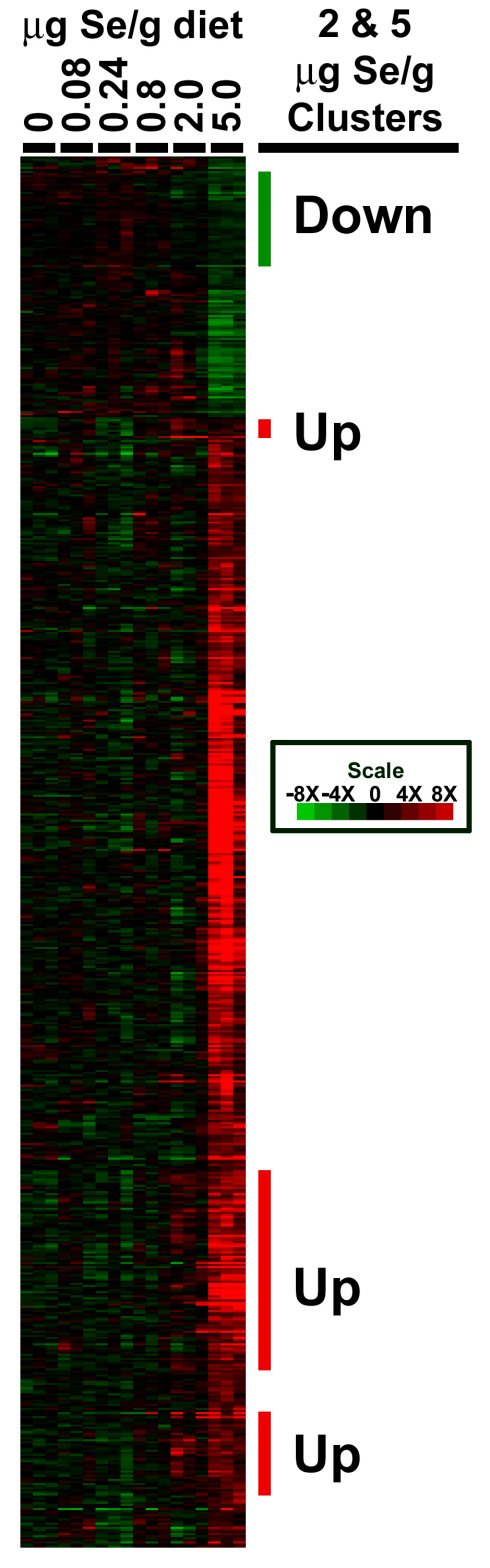
**5 μg Se/g diet elicits a unique transcriptional effect**. Unsupervised hierarchical clustering with RMA generated expression values for the 667 unique Se-specific transcripts (Figure 5). Columns represent liver expression from individual rats fed 0, 0.08, 0.24, 0.8, 2 and 5 μg Se/g diet. Rows represent probe sets. RMA gene expression is shown using the indicated pseudocolor scale from -8X (green) to +8X (red) relative to values for rats fed 0.24 μg Se/g. Clusters that are up-regulated by 2 and 5 μg Se/g diet are highlighted in red and the cluster down-regulated by 2 and 5 μg Se/g diet is highlighted in green.

Unsupervised hierarchical clustering of the Se-specific transcript expression for all 18 rat microarrays showed the unique transcriptional response in the rats fed 5 μg Se/g diet vs. lower Se intakes (Figure 6). Inspection of the resulting treeview diagram identified clusters of high Se-regulated expression in which 2 μg Se/g diet appeared to have a similar, albeit more variable effect. Three clusters containing 117 transcripts (72 genes) were up-regulated to some extent by 2 as well as 5 μg Se/g diet, and one distinct cluster containing 44 transcripts (25 genes) was down-regulated to some extent by 2 as well as 5 μg Se/g diet (Figure 6). Functional analysis revealed a set of genes within the clusters that were up-regulated by 2 and 5 μg Se/g that are involved in glucose transport, insulin signaling, or glycoprotein biosynthesis (Sort1, Sorbs1, Grb10, Star, Chsy1, St3gal2, Stxbp1, Soat1).

### Identification of High Se Molecular Biomarkers

Molecular biomarkers regulated by 2 μg Se/g may be useful in predicting high Se intake that is not yet overtly toxic. Genes regulated by 2 μg Se/g showed several unique expression patterns, which were confirmed by qRT-PCR for the genes in Table 3. Rgs4 and Ccdc80 expression as assessed by qRT-PCR were up-regulated 8-fold and 2-fold by 2 μg Se/g, respectively, but to a lesser extent of 4-fold and 1.5-fold, respectively, by 5 μg Se/g (Additional file 2, Figure S2A and S2B). Expression of Oxct1 and RGD1560666 increased in a dose-response fashion with increasing Se intake above the requirement, reaching 2-fold and 1.5-fold, respectively, by 2 μg Se/g, and 3-fold and 2-fold, respectively, by 5 μg Se/g (Additional file 2, Figure S2C and S2D). qRT-PCR, however, did not confirm that Cirbp decreases with high Se supplementation (Additional file 2, Figure S2E). In addition, several genes with marginal differential expression at 2 μg Se/g, as determined by RMA analysis, were found to be significantly up-regulated 5-fold (Timp3) or down-regulated 3-fold (RGD1564865) when analyzed using qRT-PCR (Additional file 2, Figure S2G and S2H).

**Figure 7:**
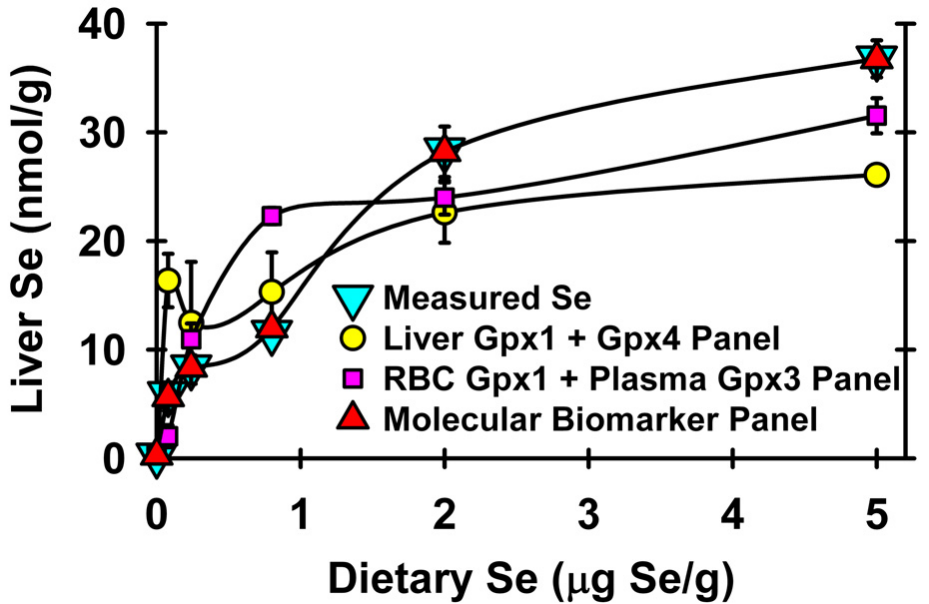
**Prediction of liver Se by biomarker panels**. Liver Se concentration activity as determined by actual measurement or calculated using biomarker panels based on liver Gpx1 plus liver Gpx4 activities, based on RBC Gpx1 plus plasma Gpx3 activities, or based on the 11-transcript molecular biomarker panel identified by multiple regression analysis, as described in the text. Values are means ± SEM.

Individual expression values for the 6 transcripts regulated by 2 μg Se/g and the 6 transcripts regulated by Se deficiency in rat study 2 were subjected to multiple regression analysis against the individual liver Se concentrations, with stepwise elimination of transcripts with non-significant correlation coefficients (P > 0.05), to yield an equation with the significant correlation coefficients that predicts liver Se concentration, as described previously [30]. This analysis resulted in a panel of 11 transcripts (6 regulated by 2 μg Se/g and 5 regulated by Se deficiency), with an overall correlation coefficient of 0.9988 (P < 10^-6^) that accounted for 99% of the variation in liver Se concentration over the full range from 0 to 5 μg Se/g diet (Figure 7). In contrast, panels based on conventional liver selenoenzyme activity biomarkers (Gpx1 plus Gpx4 activity) or blood selenoenzyme biomarkers (RBC Gpx1 plus plasma Gpx3 activity) only predicted 57% or 80%, respectively, of the variability in liver Se concentration.

## Discussion

In all 3 studies presented here, Se deficiency did not significantly affect growth, as observed in previous studies [4,5]. In rat study 2, however, a high Se intake of 5 μg Se/g diet significantly decreased growth as compared to intakes ≤ 2 μg Se/g diet. Growth defects and other symptoms of Se toxicity resulting from intakes of 3 to 5 μg Se/g have been reported in previous studies [1,31-33]. Although growth was not affected by Se deficiency, liver Se, liver and RBC Gpx1 activity, and plasma Gpx3 activity were all dramatically decreased in Se-deficient mice and rats as compared to Se-adequate animals. Gpx1 mRNA was also significantly down-regulated in Se-deficient tissues, 2.1 to 3.7 fold, and mRNA levels for several other selenoproteins were significantly down-regulated by Se deficiency, as we previously reported [8,26]. Thus, the Se-deficient animals were Se-deficient at the biochemical and molecular level, but otherwise indistinguishable from Se-adequate animals. Liver Gpx1 activity was not further increased by super-nutritional Se, and was slightly decreased by 5 μg Se/g as compared to the Se-adequate diet (0.24 μg Se/g), most likely due to liver damage.

Microarray analysis of >30,000 transcripts in the liver of rats fed Se deficient to 5 μg Se/g diet found that an intake of 5 μg Se/g is required to alter the expression of a large set of transcripts. This is the first report of a large and distinct transcriptional response profile induced by Se toxicity. In contrast, Se intakes less than 5 μg Se/g diet significantly changed < 10 transcripts as compared to a Se-adequate intake within an experiment. The distinct transcriptional effect of 5 μg Se/g correlates with growth and ALT activity, the other markers of toxicity measured in this study. The vastly expanded number of gene expression changes observed in this study thus is a newly-identified marker of Se toxicity. Importantly, as many as half of the 1193 transcripts altered by the 5 μg Se/g diet treatment may be Se-specific, as 715 transcripts still remained after removing transcripts responding to general toxicity and caloric restriction.

Previous microarray studies have investigated the transcriptional effects of Se supplementation up to 1.0 μg Se/g diet in rodents, of up to 200 μg Se/day in human subjects, and of up to 22 μM Se in cultured cells. A search of the Se-regulated genes in 9 previous Se microarray studies [11-18,34] found only a few genes that were identified in more than 1 study. The only genes regulated by >3 studies, however, were selenoproteins, suggesting that most of the genes identified were not responding specifically to high Se. Many of these studies are complicated by the fact that high Se treatments were only compared to Se-deficient treatments, so that many of the transcriptional changes are likely to be the result of Se deficiency instead of Se supplementation. One study that did compare a high Se treatment to a Se-adequate treatment (1.0 μg Se/g vs. 0.2 μg Se/g) [13], found no significantly altered genes. Based on the present study, it is very likely that the lack of Se-specific regulation in these previous studies is due to insufficient Se to cause large, significant transcriptional responses. Additional studies with near toxic Se levels may be necessary to determine whether the transcripts identified in the present study are truly Se-specific.

The levels of high Se in the present studies (8, 20 and 50-times the Se requirement) are relevant to high Se intakes in humans. A study of Chinese subjects living in a high Se area of China found no adverse effects of Se at intakes of 800 μg/day (16X requirement) [35]. A small supplementation trial in men with prostate cancer gave subjects 1600 or 3200 μg Se/d as selenized yeast (32X and 64X requirement) for about 12 months and found no observable adverse effects in the group supplemented with 1600 μg Se/d [36]. The subjects supplemented with 3200 μg Se/d, however, reported symptoms of Se toxicity, and a few men reached plasma Se levels exceeding 1000 ng/ml, which was reported to be the threshold for the onset of Se toxicity in the Chinese study [35]. These reports in humans agree with the observations from the present study of no adverse effects of intakes up to 20-times the requirement and significant signs of Se toxicity at intakes ≥50-times the requirement. Additionally, the microarray data presented here indicates that super-nutritional Se intakes 8 to 20-times the Se requirement are not sufficient to cause a large transcriptional response. This is an important point because levels used in cancer prevention trials are typically 200 μg/day, or 4-times the requirement, which will bring total Se intake to 5 to 8-times the requirement depending on dietary Se intake from foods. This data thus suggest that cancer prevention associated with super-nutritional Se supplementation may not be mediated by transcriptional changes.

Se deficiency highly regulates the expression of some, but not all selenoprotein mRNAs [5,8,26,37]. Most notable of the regulated selenoprotein genes is Gpx1, with its expression dropping to < 10% of Se-adequate levels in the rat model. Our recent studies have shown that in addition to Gpx1, Sepw1 and Selh are also highly regulated by Se deficiency and several other selenoprotein mRNAs are moderately regulated [8,26]. Interestingly, there were no additional non-selenoprotein genes found to be down-regulated by Se deficiency, reinforcing the Se-specificity of selenoprotein regulation. Importantly, both microarray and qRT-PCR expression of the genes up-regulated in Se deficiency show that like selenoprotein mRNAs, these genes are restored to adequate levels in the mice fed a Se marginal diet (0.05 μg Se/g diet) (Additional file 1, Figure S1).

A small set of genes were found to be significantly and consistently up-regulated by Se deficiency. Comparison of these genes with a dataset of 21 well-characterized Nrf2-targeted genes containing Nrf2-binding sites [38] plus a dataset of 1055 Nrf2-targeted genes identified recently by ChiP analysis [39] revealed that the majority of the genes up-regulated by Se deficiency were Nrf2 targets (Table 1). NADPH dehydrogenase quinone 1 (Nqo1), a classic target of Nrf2 [40,41], did not reach significance in this study, but was up-regulated 1.5 to 2 fold by Se-deficient treatments. Similarly, ABC transporters (also known as multidrug resistance proteins) and glutathione S-transferases, are known targets of Nrf2 regulation [42,43], and were consistently up-regulated by Se deficiency. Two of the up-regulated ABC transporters, Abcc4 and Abcc3, are reported to be involved in detoxification of a variety of drugs including acetaminophen [44]. In comparison, the only gene down-regulated by Se-deficiency and overlapping with these Nrf2 datasets was thioredoxin reductase-1 in mouse liver (Table 2). Two additional Nrf2 target genes, Rgs4 and Tsku, were also found to be up-regulated by 2 μg Se/g diet (Table 3). Overlap analysis of the 1193 transcripts that were significantly altered by 5 μg Se/g diet and the Nrf2-regulated gene datasets identified 99 Nrf2 targets that were differentially-regulated in Se toxicity (Additional file 3, Table S1), including 49 genes retained in the Se-specific dataset. The prevalence of Nrf2-regulated genes in genes significantly altered by Se toxicity as well as up-regulated by Se deficiency indicates that Se excess as well as Se deficiency increases oxidative stress.

Functional analysis of the genes in the Se-specific dataset indicates their involvement in processes such as cell movement/morphogenesis and development/angiogenesis (Table 4). ECM-related genes were particularly affected by Se toxicity. For example, collagen fibril organization was enriched by a factor of 6.72 (6 of 23 total genes present in the Se-specific set, FDR = 0.006). The genes in this category included four collagen genes (Col12a1, Col5a2, Col1a2, Col11a1), one serine proteinase inhibitor (Serpinh1) and one annexin (Anxa2). Further searching of the Se-specific dataset found a total of seven collagen genes. In addition, many other ECM-related genes were present in the 5 μg Se/g dataset. Collagen genes were also reported to be regulated by Se-methylselenocysteine in a prostate cancer cell line (LNCaP), but only one of these (Col4a5) was regulated in the same direction in the present study [17]. Additional evidence for an association between Se and collagen metabolism comes from a study which found that Se supplementation of rats with 0.3 μg Se/g diet for 10 weeks increased collagen content of the skin, but decreased it in other tissues including liver [45]. The regulation of ECM components may also be relevant to the anti-carcinogenic effect of Se as the ECM plays an important role in cell migration and tumor progression. In addition, collagen-regulation by Se may underlie some physiologic effects of Se toxicity, as the symptoms of Se toxicity include changes or malformations in tissues comprised primarily of collagen, nails and hair in humans and hooves in grazing animals [9,46].

Genes related to glucose metabolism were enriched in the clusters that were up-regulated to some extent by 2 as well as 5 μg Se/g diet. There is some evidence that high Se status is related to diabetes and it is known that a selenosugar is one of the major excretory metabolites for Se [24,25,47]. Se-regulation of glucose-related genes may be another piece of the puzzle linking Se and glucose metabolism.

We also conducted GOMiner analysis of the genes that were removed from the original 1193-transcript toxic Se dataset due to overlap with genes altered by general toxicity and/or calorie restriction. This analysis identified 90 significantly enriched (False Discovery Rate = FDR < 0.05) biological processes that were not specifically associated with Se toxicity; these processes appeared to be more varied than those found in the Se-specific dataset (Additional file 4, Table S2).

Very few transcripts were significantly regulated by the sub-toxic intake of 2 μg Se/g diet, but there were several clusters of transcripts altered significantly by 5 μg Se/g which were also variably affected by 2 μg Se/g diet. Two of the conventional biomarkers, growth and ALT activity, suggest 5 but not 2 μg Se/g diet is toxic. AST activity, however, suggests that high Se intake at 2 μg Se/g as well as in Se deficiency is also just beginning to cause some liver damage. Previous studies have reported adverse effects, such as reduced body weight and liver damage, from Se intakes as low as 3 μg Se/g [31]. Further studies are necessary to determine whether the transcripts within the clusters similarly regulated by 2 and 5 μg Se/g diet would gain significance and correlate with other markers of Se toxicity if studied at intakes between 2 and 5 μg Se/g diet. As 2 μg Se/g diet borders on a toxic Se intake, the 6 transcripts that were significantly regulated by this treatment may prove to be useful biomarkers of high Se intake before adverse effects are observed.

To illustrate the potential utility of a small set of high Se regulated transcripts as biomarkers of Se status, these transcripts and those regulated by Se deficiency were used successfully to predict liver Se concentration. Liver Se concentration was chosen as a marker of Se status for this example because it represents a pool of Se that continues to increase above the Se requirement, similar to hypothetical pools of Se associated with the anticarcinogenic activity of Se. The resulting 11-gene biomarker panel accounted for 99% of the variability in liver Se concentration across the range from Se-deficient to toxic Se, illustrating the potential of molecular biomarkers to predict Se status. Comparison of the molecular biomarker panel curve in Figure 7 with the conventional biomarker curves shown in Figure 2B-D clearly illustrates why these conventional biomarkers are ineffective for assessment of high Se status.

The high Se regulated transcripts identified in this study were associated with high Se intake in rats, but these studies did not show that these changes are specific for super-nutritional or toxic Se status. The use of transcript datasets associated with general toxicity or calorie restriction reduced the dataset to 667 unique transcripts which were more likely to be Se-specific, but, as shown by the variety of biological processes enriched in this dataset (Table 4), it is clear that most of these transcripts are likely to be altered by a variety of conditions, not just high Se. The molecular biomarker panel, however, illustrates that it may be possible to identify panels of these transcripts that collectively can be specific for a condition such as high Se status. Additional studies will be needed to further refine the sets of Se-specific transcripts that can be used as biomarkers and to uncover genes and processes associated with Se homeostasis and the effects of high Se supplementation.

## Conclusion

This study showed that a Se intake which affected conventional markers of toxicity in rats (5 μg Se/g diet) also altered the expression of 1193 transcripts when compared to Se-adequate rats. High, but non-toxic Se intakes (0.8 and 2 μg Se/g diet), however, produced very few expression changes, providing the first evidence that a vastly expanded number of transcriptional changes is a biomarker of Se toxicity. Over half of the transcripts regulated by Se toxicity may be Se-specific, as 715 transcripts still remained after removing those responding to general toxicity and caloric restriction. Genes that respond specifically to Se toxicity are enriched in processes related to the extracellular matrix, including a number of collagen genes. In addition to down-regulating selenoprotein genes, Se deficiency consistently up-regulated several genes known to be targets of Nrf2 regulation. The small set of transcripts significantly regulated by 2 μg Se/g diet combined with those regulated by Se deficiency was used to identify a biomarker panel that over the range from Se-deficient to 50-times the requirement could account for 99% of the variability in liver Se concentration. Further studies at Se intakes between 2 and 5 μg Se/g diet will be needed to better define the association between transcriptional changes and Se toxicity. In addition, microarray studies on toxic Se intakes with other forms of Se such as selenomethionine, high Se yeast and selenate may help to identify regulated transcripts that are specific for different forms of Se and ideally even to identify biomarkers specific for high Se status regardless of Se form.

## Methods

### Reagents

Molecular biology reagents were purchased from Promega (Madison, WI), Invitrogen (Carlsbad, CA) or Sigma (St. Louis, MO). All other chemicals were of molecular biology or reagent grade.

### Animals and Diets

#### Mouse Study

Male mouse pups from Se-adequate dams in our wild-type colony (predominately C57 black background) were weaned 18 days after birth and housed individually in hanging-wire cages, as described previously in detail [26]. The basal Se-deficient diet was a torula-yeast diet containing 0.005 μg Se/g by analysis, supplemented with 100 mg/kg all-rac-α-tocopherol acetate and 0.4% L-methionine to prevent liver necrosis and ensure adequate growth, as described previously [4]. Mice were fed the basal diet supplemented with 0, 0.05, or 0.2 μg Se/g as Na_2_SeO_3 _for 35 days (n = 3/group) to provide Se-deficient, Se-marginal or Se-adequate diets.

#### Rat Studies 1 and 2

Male, 21-day old weanling rats were obtained form Holtzman (Madison, WI) and were housed individually in hanging-wire cages. Study 1 rats were fed the basal Se-deficient diet supplemented with graded levels of Se: 0, 0.016, 0.04, 0.06, 0.08, 0.12, 0.16, 0.24, 0.4 or 0.8 μg Se/g diet as Na_2_SeO_3 _for 28 days (n = 6/group), as described previously in detail [8]. Study 2 rats were fed the basal diet supplemented with 0, 0.08, 0.24, 0.8, 2 or 5 μg Se/g diet (n = 4/group) for 28 days. All animals had free access to food and water and body weight was measured bi-weekly. The care and treatment protocols were approved by the University of Wisconsin Institutional Animal Care and Use Committee.

### Enzyme Activity Analysis

Tissue supernatants were prepared by homogenizing in 9 volumes of sucrose buffer [20 mM Tris/HCl, pH 7.4, 0.25 M sucrose, 1 mM EDTA and 0.1% peroxide-free Triton X-100]. Homogenates were centrifuged at 10,000 g for 15 min at 4°C, model J2-21M, JA-21 rotor (Beckman Instruments, Palo Alto, CA). Gpx1 activity in liver, kidney and red blood cells and Gpx3 activity in plasma were measured by the coupled assay procedure using 120 μM H_2_O_2 _[48]. Gpx4 activity was measured by the coupled assay procedure using 78 μM phosphatidylcholine hydroperoxide, its specific substrate [5]. For both assays, 1 unit is the amount of enzyme that will oxidize one μmole of GSH per min under these conditions. Alanine aminotransferase (ALT) and aspartate aminotransferase (AST) activity were measured in study 2 rat plasma using kits #318 and #319, respectively, following the manufacturer's protocol (Genzyme Diagnostics, Charlottetown PE, Canada). The protein concentrations of samples were determined by the method of Lowry et al. [49]. Neutron activation analysis was kindly conducted by the University of Missouri Research Reactor to determine liver and diet Se concentrations [50]. NIST SRM 1577 Bovine Liver was used as a certified quality control sample for the liver analyses.

### RNA isolation and microarray processing

Total RNA from mouse liver and kidney (n = 3/diet group) was isolated with TRIzol Reagent (Invitrogen, Carlsbad, CA) following the manufacturer's protocol. Subsequently, mRNA was purified using an oligo-dT-linked Oligotex resin (Qiagen, Valencia, CA). The resulting mRNA (1 μg) was used to synthesize double stranded cDNA [51], which was then purified and used for in vitro transcription of biotin-labeled cRNA with the Enzo BioArray HighYield RNATranscript Labeling Kit. The cRNA was purified using an RNeasy kit (Qiagen) and fragmented at 94°C for 35 min in a buffer containing 200 mM Tris-Acetate, pH 8.0, 500 mM potassium acetate, 150 mM magnesium acetate. Integrity and size of total RNA, cRNA and fragmented cRNA for each sample was determined by formaldehyde-agarose gel electrophoresis. RNA or DNA obtained at each step was quantitated using a ND-1000 UV-Vis Spectrophotometer (NanoDrop Technologies, Wilmington, DE). The mouse study used GeneChip Mouse Genome 430 2.0 arrays (Affymetrix, Santa Clara, CA), which contain over 45,000 probe sets that target transcripts that represent over 34,000 mouse genes. Fragmented cRNAs and GeneChip arrays were submitted to the Gene Expression Center at UW-Madison for hybridization, washing and scanning following standard manufacturer's protocols.

Total RNA from rat liver was isolated as described above and was then submitted to the Gene Expression Center at UW-Madison for processing to cRNA, hybridization, washing and scanning. The rat studies used GeneChip Rat Genome 230 2.0 arrays (Affymetrix), which contain over 31,000 probe sets that target transcripts representing over 28,700 rat genes.

### Microarray analysis

Robust Multichip Averaging (RMA) was used to correct for background, normalize and generate expression data [52]. The .CEL files for each microarray study were analyzed with the affy package within the Bioconductor open-source project using 'R' statistical software [53]. Arrays were processed separately for each study and for each tissue within the mouse study. The Limma (Linear Models for Microarray Data) package was then used to identify differentially expressed transcripts from the RMA normalized data [54]. This package uses linear models and Bayesian statistics to test the variance in expression of each probe set on the array and P-values are then adjusted for multiple testing. For each analysis a contrast matrix was designed to identify differentially expressed probe sets in each Se treatment group as compared to the Se-adequate group (0.2 μg Se/g diet for mice and 0.24 μg Se/g diet for rats). The arrays from rat study 1 were also analyzed separately using RMA and Limma to compare expression in 0.8 vs. 0.08 μg Se/g diet rats. A probe set was considered differentially expressed if the adjusted P-value was < 0.05. The microarray data discussed in this publication have been deposited in NCBI's Gene Expression Omnibus [55] and are accessible through GEO Series accession number GSE23895

http://www.ncbi.nlm.nih.gov/geo/query/acc.cgi?acc=GSE23895.

### qRT-PCR analysis

Selected genes found to be differentially expressed by microarray were validated with qRT-PCR as previously described [26]. Relative expression was calculated using the method of Pfaffl [56], normalized to the mean of control genes Actb and Gapdh and set relative to the mean of Se-adequate animals. qRT-PCR and RMA expression data are presented as mean " SEM (n = 3/diet group).

### Transcript Filtering, Clustering and Functional Analysis

Transcripts that were significantly regulated by Se toxicity (5 μg Se/g diet) were filtered to obtain a set of Se-specific response transcripts. Transcripts in the Se toxicity set were compared to transcripts on the RatToxFX 1.0 array (Affymetrix) and to probe sets altered by caloric restriction in a recent study by Pohjanvirta et al. [29]. Caloric restriction microarray data was downloaded from the Gene Expression Omnibus at NCBI (GEO accession number = GSE9121) and independently analyzed from the treatment restricting feed intake in rats for 10 days vs. control rats. The probe sets found to overlap between the Se toxicity set and these datasets were then removed to enrich the data with Se-specific transcripts. Additionally, redundant probe sets representing genes found to overlap between datasets were removed from the Se toxicity set. Hierarchical clustering was performed on the remaining transcripts using RMA-calculated expression and visualization of the resulting tree was done with Treeview software [57]. The GOMiner tool was used to identify biological processes that were significantly enriched in the particular datasets vs. all genes on the Rat 230 2.0 Genome array [58]. A biological process was considered significantly enriched if the false discovery rate (FDR) was less than 0.05.

### Multiple Regression Analysis

Microarray expression values for potential biomarker genes were regressed against corresponding liver Se concentrations using the Excel 2007 Data Analysis add-in, as described previously [30]. Biomarkers with non-significant coefficients were removed in a step-wise fashion until all remaining biomarkers in the panel had significant coefficients (P < 0.05).

### Statistics

Animal growth, Se concentrations, enzyme activities and relative expression data are presented as mean ± SEM. For the mouse study and rat study 1, n = 3/group. For rat study 2, n = 3 or 4/group. All data were analyzed by ANOVA using a fixed model testing the main effect of diet. Significant differences between means were assessed by Duncan's multiple range analysis (P < 0.05) [59].

## Abbreviations

ALT: alanine aminotransferase; AST: aspartate aminotransferase; CalRestr: calorie restriction; ECM: extra cellular matrix; FDR: False Discovery Rate; GO: gene ontology; Gpx1: glutathione peroxidase-1; Gpx3: glutathione peroxidase-3; Limma: Linear Models for Microarray Data; NHANES: National health and nutrition examination survey; NPCT: National Prevention of Cancer Trial; Nqo1: NAD(P)H dehydrogenase, quinone 1; qRT-PCR: quantitative real time polymerase chain reaction; RBC: red blood cell; RDA: Recommended dietary allowance; RMA: robust multichip averaging; Se: selenium; SELECT: Selenium and Vitamin E Cancer Prevention Trial; UL: tolerable upper intake level.

## Authors' contributions

AMR conducted the research work, analyzed the data, and wrote and edited the manuscript. RAS was responsible for overseeing experimental design and for manuscript editing and preparation. Both authors read and approved the final manuscript.

## Supplementary Material

Additional file 1 **Supplemental Figure S1**. qRT-PCR and microarray expression for selected genes up-regulated by Se-deficiency. Click here for file

Additional file 2 **Supplemental Figure S2**. qRT-PCR and microarray expression for genes regulated by 2 μg Se/g diet. Click here for file

Additional file 3 **Supplemental Table S1**. List of known Nrf2-regulated transcripts with altered expression in rats fed 5 μg Se/g diet. Click here for file

Additional file 4 **Supplemental Table S2**. Full list of biological processes enriched in genes removed from the original toxic Se data set due to overlap with genes altered by general toxicity and/or calorie restriction. Click here for file
