# Supplementary material for: Selenium toxicity but not deficient or super-nutritional selenium status vastly alters the transcriptome in rodents
Source: BMC Genomics. 2011 Jan 12;12:26. doi: 10.1186/1471-2164-12-26 (PMC3032699; doi:10.1186/1471-2164-12-26)
Supplement: Additional file 1 — Supplemental Figure S1. qRT-PCR and microarray expression for selected genes up-regulated by Se-deficiency. [file 1471-2164-12-26-S1.PDF]

Raines & Sunde, Supplemental Figure 1

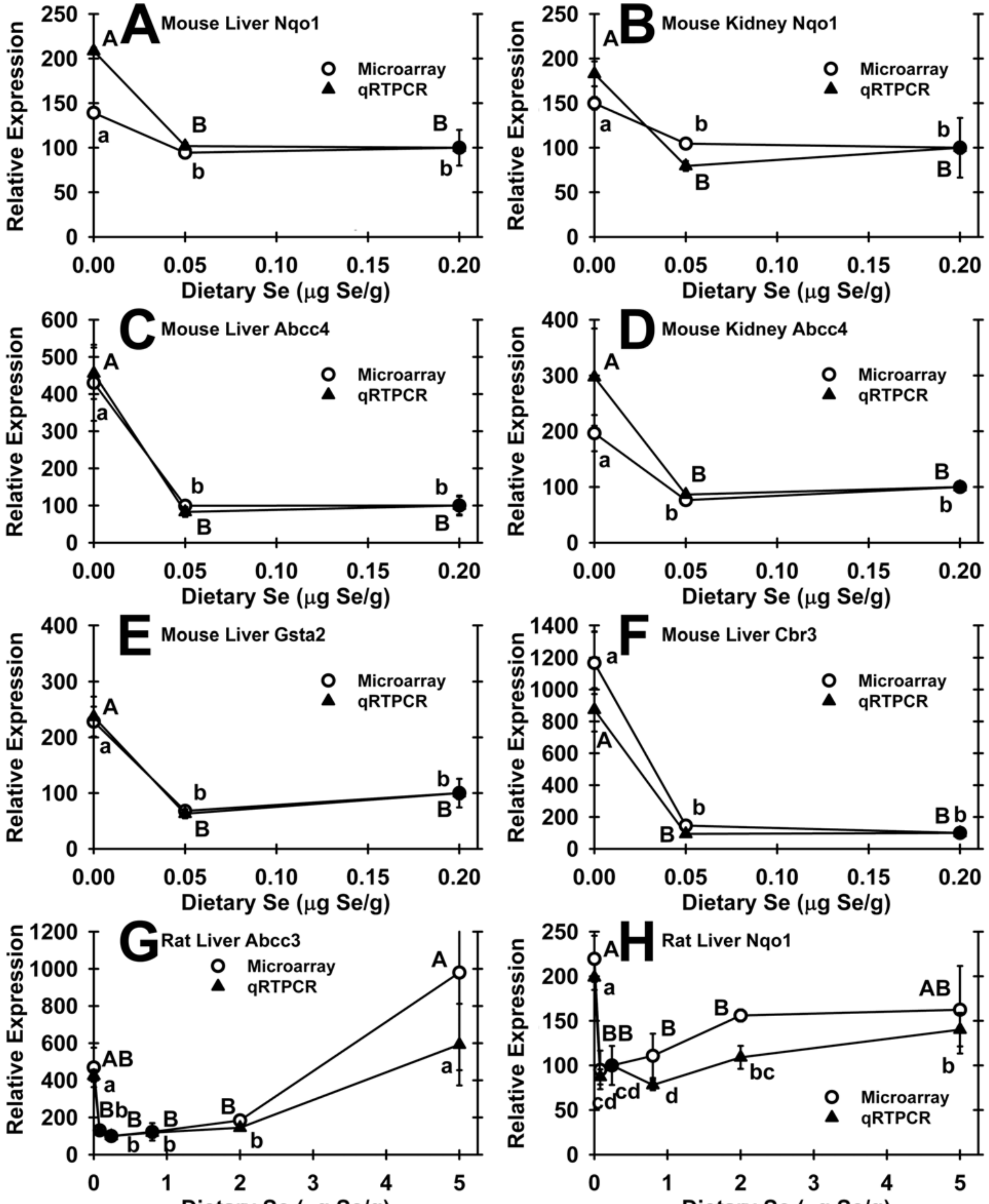

Supplemental Figure 1. qRT-PCR and microarray expression for selected genes up-regulated by Se-deficiency. Relative mRNA levels for Nqo1 (A, B), Abcc4 (C, D), Gsta2 (E), and Cbr3 (F) in mice fed diets supplemented with 0 to 0.2 µg Se/g, and for Abcc3 (G) and Nqo1 (H) in rats fed diets supplemented with 0 to 5 µg Se/g, as determined by microarray and qRT-PCR on total RNA from the indicated tissues. Microarray values are means $\pm$ SEM (n=3) of RMA generated expression values. qRT-PCR values were determined in triplicate for each sample, normalized to the mean of Gapdh and Actb mRNA levels in each sample, expressed as a percentage of Se-adequate (0.2 µg Se/g, mice; 0.24 µg Se/g, rats) levels, and plotted as means $\pm$ SEM (n=3). The effect of dietary Se was significant ($P<0.05$) for all response curves; values with a common letter are not significantly different ($P\geq0.05$).
