# Supplementary material for: Selenium toxicity but not deficient or super-nutritional selenium status vastly alters the transcriptome in rodents
Source: BMC Genomics. 2011 Jan 12;12:26. doi: 10.1186/1471-2164-12-26 (PMC3032699; doi:10.1186/1471-2164-12-26)
Supplement: Additional file 2 — Supplemental Figure S2. qRT-PCR and microarray expression for genes regulated by 2 μg Se/g diet. [file 1471-2164-12-26-S2.PDF]

Raines & Sunde, Supplemental Figure 2 A-F

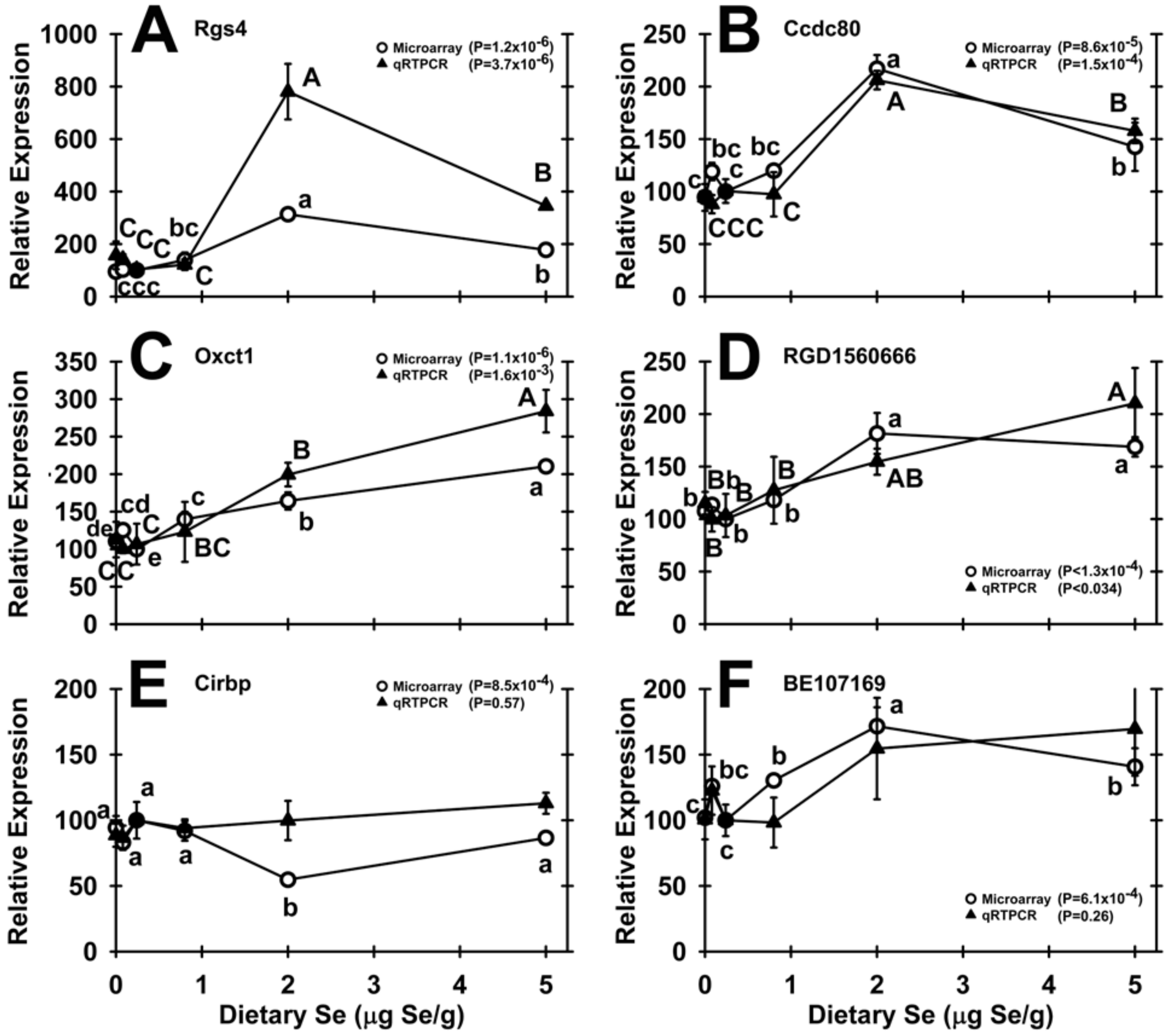

Supplemental Figure 2. qRT-PCR and microarray expression for selected genes regulated by 2 μg Se/g. Relative mRNA levels for Rgs4 (A), Ccdc80 (B), Oxct1 (C), RGD1560666 (D), Cirbp (E), BE107169 (F), Timp3 (G), RGD15644865 (H), Tsku (I), AA945268 (J), and Hsph1 (K) in total liver RNA from rats fed diets supplemented with 0 to 5 μg Se/g, determined as described in Supplemental Figure 1. Values are means ± SEM (n =3) of RMA generated expression values or qRT-PCR values. Indicated P-values were determined by ANOVA; values with a common letter are not significantly different (P≥0.05) as determined by multiple range testing for datasets with P<0.05 by ANOVA.

Raines & Sunde, Supplemental Figure 2 G-K

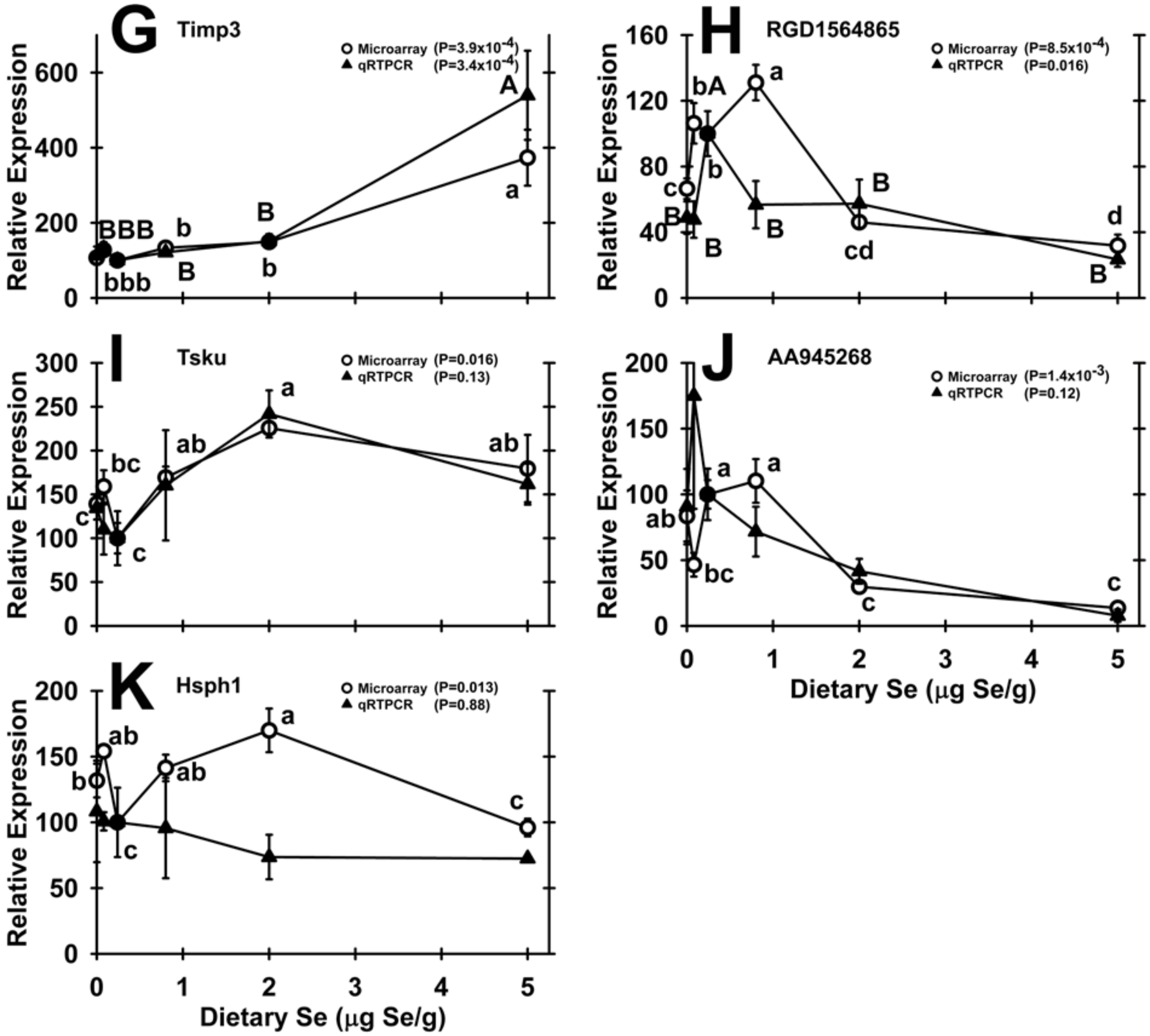

Supplemental Figure 2. qRT-PCR and microarray expression for selected genes regulated by 2 µg Se/g. Relative mRNA levels for Rgs4 (A), Ccdc80 (B), Oxct1 (C), RGD1560666 (D), Cirbp (E), BE107169 (F), Timp3 (G), RGD15644865 (H), Tsku (I), AA945268 (J), and Hsph1 (K) in total liver RNA from rats fed diets supplemented with 0 to 5 µg Se/g, determined as described in Supplemental Figure 1. Values are means $\pm$ SEM (n =3) of RMA generated expression values or qRT-PCR values. Indicated P-values were determined by ANOVA; values with a common letter are not significantly different ($P \geq 0.05$) as determined by multiple range testing for datasets with $P<0.05$ by ANOVA.
