# Supplementary material for: Selenium toxicity but not deficient or super-nutritional selenium status vastly alters the transcriptome in rodents
Source: BMC Genomics. 2011 Jan 12;12:26. doi: 10.1186/1471-2164-12-26 (PMC3032699; doi:10.1186/1471-2164-12-26)
Supplement: Additional file 3 — Supplemental Table S1. List of known Nrf2-regulated transcripts with altered expression in rats fed 5 μg Se/g diet. [file 1471-2164-12-26-S3.PDF]

Supplemental Table S1: Nrf1-targets regulated by 5 μg Se/g diet

Genbank Accession	Gene Symbol	Gene Title	Fold Change[1]	Adj P-value[2]	Se Specific[3]
NM_012577	Gstp1	glutathione S-transferase pi 1	5.66	0.001	
NM_001107673	Slc7a11	solute carrier family 7 (cationic amino acid transporter, y+	5.66	0.000	
NM_080581	Abcc3	ATP-binding cassette, sub-family C (CFTR/MRP), member 3	5.28	0.001	
NM_019371	Egln3	EGL nine homolog 3 (C. elegans)	4.59	0.030	
NM_020540	Gstm3	glutathione S-transferase mu 3	4.59	0.005	
NM_001135009	Col4a1	collagen, type IV, alpha 1	4.00	0.000	
NM_031764	Ddr2	discoidin domain receptor tyrosine kinase 2	4.00	0.000	
NM_012598	Lpl	lipoprotein lipase	4.00	0.018	
NM_001003401	Enc1	ectodermal-neural cortex 1	3.73	0.005	
NM_031588	Nrg1	neuregulin 1	3.73	0.000	
NM_053861	Tnc	tenascin C	3.48	0.001	
NM_147210	Nr1d2	nuclear receptor subfamily 1, group D, member 2	3.25	0.005	
NM_001034952	Pqlc3	PQ loop repeat containing 3	3.25	0.000	X
NM_053713	Klf4	Kruppel-like factor 4 (gut)	3.03	0.004	X
NM_001008562	Lmcd1	LIM and cysteine-rich domains 1	3.03	0.005	
NM_053018	Cd9	CD9 molecule	2.83	0.010	X
NM_053356	Col1a2	collagen, type I, alpha 2	2.83	0.007	X
NM_001109093	Grb10	growth factor receptor bound protein 10	2.83	0.000	X
NM_001107635	Lama4	laminin, alpha 4	2.83	0.000	X
NM_001030020	Prkar2b	protein kinase, cAMP dependent regulatory, type II beta	2.83	0.000	X
NM_133411	Abcc4	ATP-binding cassette, sub-family C (CFTR/MRP), member 4	2.64	0.001	
NM_031642	Klf6	Kruppel-like factor 6	2.64	0.000	X
NM_053297	Pkm2	pyruvate kinase, muscle	2.64	0.001	
NM_012755	Fyn	FYN oncogene related to SRC, FGR, YES	2.46	0.000	X
NM_001007554	Fblim1	filamin binding LIM protein 1	2.30	0.002	X
NM_019337	Rgs10	regulator of G-protein signaling 10	2.30	0.007	
NM_031544	Ampd3	adenosine monophosphate deaminase 3	2.14	0.000	X
NM_001039002	Fam43a	family with sequence similarity 43, member A	2.14	0.006	
NM_001007712	Sdpr	serum deprivation response	2.14	0.006	X
NM_001108080	Spic	Spi-C transcription factor (Spi-1/PU.1 related)	2.14	0.025	X
NM_053369	Tcf4	transcription factor 4	2.14	0.039	X
NM_001034068	Tpm1	tropomyosin 1, alpha	2.14	0.002	X
NM_012620	Serpine1	serine (or cysteine) peptidase inhibitor, clade E, member 1	2.00	0.003	
NM_053424	Slc4a4	solute carrier family 4 (anion exchanger), member 4	2.00	0.036	

NM_031695	St3gal2	ST3 beta-galactoside alpha-2,3-sialyltransferase 2	1.99	0.001	X
XM_574285	Ncoa7	nuclear receptor coactivator 7	1.96	0.015	X
NM_012904	Anxa1	annexin A1	1.93	0.030	X
NM_080771	Inhbb	inhibin beta-B	1.93	0.010	
NM_022526	Dap	death-associated protein	1.89	0.022	
NM_012827	Bmp4	bone morphogenetic protein 4	1.88	0.020	
NM_001130573	Maff	v-maf musculoaponeurotic fibrosarcoma oncogene homolog F (av	1.87	0.001	
NM_001047858	Srxn1	sulfiredoxin 1 homolog (S. cerevisiae)	1.87	0.015	X
NM_013107	Bmp6	bone morphogenetic protein 6	1.85	0.001	
NM_057211	Klf9	Kruppel-like factor 9	1.85	0.011	
NM_012580	Hmox1	heme oxygenase (decycling) 1	1.84	0.033	
NM_053966	Lamc1	laminin, gamma 1	1.82	0.001	
NM_001191705	Fat4	FAT tumor suppressor homolog 4 (Drosophila)	1.77	0.047	X
NM_017214	Rgs4	regulator of G-protein signaling 4	1.77	0.018	X
NM_001109255	Sspn	sarcospan	1.77	0.013	X
NM_022294	Eltd1	EGF, latrophilin and seven transmembrane domain containing 1	1.67	0.019	X
NM_001108009	Rasgrp3	RAS guanyl releasing protein 3 (calcium and DAG-regulated)	1.65	0.042	
NM_001015020	Tgif1	TGFB-induced factor homeobox 1	1.65	0.006	X
NM_022946	Dlgap1	discs, large (Drosophila) homolog-associated protein 1	1.61	0.005	X
NM_139252	Ppap2c	phosphatidic acid phosphatase type 2c	1.61	0.041	
NM_001005547	Tspan3	tetraspanin 3	1.61	0.017	X
NM_022407	Aldh1a1	aldehyde dehydrogenase 1 family, member A1	1.59	0.012	
NM_001169103	Crim1	cysteine rich transmembrane BMP regulator 1 (chordin like)	1.59	0.040	
M_054011	Sh3bp5	SH3-domain binding protein 5 (BTK-associated)	1.58	0.048	X
NM_001012044	Lcp1	lymphocyte cytosolic protein 1	1.56	0.024	
NM_013194	Myh9	myosin, heavy chain 9, non-muscle	1.56	0.023	
NM_001191560	Atg16l2	ATG16 autophagy related 16-like 2 (S. cerevisiae)	1.55	0.004	X
NM_033485	Pawr	PRKC, apoptosis, WT1, regulator	1.54	0.029	X
NM_138914	Fnbp1	formin binding protein 1	1.53	0.014	
NM_001134727	Lrch1	leucine-rich repeats and calponin homology (CH) domain conta	1.52	0.011	X
NM_001007732	Serpinb9	serine (or cysteine) peptidase inhibitor, clade B, member 9	1.52	0.018	X
NM_053653	Vegfc	vascular endothelial growth factor C	1.52	0.006	X
NM_001005265	Cr1l	complement component (3b/4b) receptor 1-like	1.49	0.009	X
NM_001108099	Mdm2	Mdm2 p53 binding protein homolog (mouse)	1.49	0.009	
NM_001171177	Tmtc2	transmembrane and	1.49	0.038	X

		tetratricopeptide repeat containing 2			
NM_031118	Soat1	sterol O-acyltransferase 1	1.48	0.016	X
NM_00116410	Add3	adducin 3 (gamma)	1.44	0.010	X
NM_001108422	Plxdc2	plexin domain containing 2	1.43	0.043	X
NM_001106268	Chsy1	chondroitin sulfate synthase 1	1.42	0.049	X
NM_001044294	Gabarapl1	GABA(A) receptor-associated protein like 1	1.41	0.041	
NM_001130540	Ext1	exostoses (multiple) 1	1.39	0.018	
NM_013057	F3	coagulation factor III (thromboplastin, tissue factor)	1.39	0.008	X
NM_030829	Grk5	G protein-coupled receptor kinase 5	1.36	0.033	X
NM_133610	Kcnh5	potassium voltage-gated channel, subfamily H (eag-related),	-1.29	0.032	X
NM_001012183	Cryz	crystallin, zeta	-1.36	0.028	X
NM_139253	Mcfd2	multiple coagulation factor deficiency 2	-1.38	0.026	
NM_133398	Mtdh	metadherin	-1.46	0.046	
NM_012592	Ivd	isovaleryl coenzyme A dehydrogenase	-1.47	0.049	X
NM_001110860	Crem	cAMP responsive element modulator	-1.69	0.001	
NM_178091	Insig2	insulin induced gene 2	-1.69	0.018	
NM_024355	Axin2	axin2	-1.74	0.028	
NM_013141	Ppard	peroxisome proliferator-activated receptor delta	-1.75	0.007	X
NM_012870	Tnfrsf11b	tumor necrosis factor receptor superfamily, member 11b	-1.82	0.019	
NM_001108358	Lnx1	ligand of numb-protein X 1	-1.84	0.003	X
NM_001107753	Lrrc4c	leucine rich repeat containing 4C	-1.88	0.005	X
NM_012567	Gja1	gap junction protein, alpha 1	-1.91	0.007	
NM_001037200	Fam82a1	family with sequence similarity 82, member A1	-1.95	0.048	
NM_001130548	Col14a1	collagen, type XIV, alpha 1	-2.00	0.033	
NM_022249	Khdrbs3	KH domain containing, RNA binding, signal transduction assoc	-2.00	0.020	X
NM_053019	Avpr1a	arginine vasopressin receptor 1A	-2.14	0.025	
NM_022521	Oat	ornithine aminotransferase (gyrate atrophy)	-2.30	0.016	
NM_012880	Sod3	superoxide dismutase 3, extracellular	-2.46	0.048	
NM_012588	Igfbp3	insulin-like growth factor binding protein 3	-2.64	0.003	
NM_001014166	Il33	interleukin 33	-2.64	0.001	
NM_053882	Ecm1	extracellular matrix protein 1	-4.92	0.000	

Nrf2-regulated transcripts in liver that were significantly regulated in 5 μg Se/g diet rats as compared to Se-adequate rats (0.24 μg Se/g diet). [1]Fold changes determined by analysis of RMA expression data with the Limma package in R software, [2]P-values were adjusted for multiple testing, [3]Genes specifically regulated by Se, determined as Se-regulated genes that did not overlap with genes altered by general toxicity and/or calorie restriction.
