# Supplementary material for: Selenium toxicity but not deficient or super-nutritional selenium status vastly alters the transcriptome in rodents
Source: BMC Genomics. 2011 Jan 12;12:26. doi: 10.1186/1471-2164-12-26 (PMC3032699; doi:10.1186/1471-2164-12-26)
Supplement: Additional file 4 — Supplemental Table S2. Full list of biological processes enriched in genes removed from the original toxic Se data set due to overlap with genes altered by general toxicity and/or calorie restriction. [file 1471-2164-12-26-S4.PDF]

Supplemental Table S2. Biological Processes enriched in genes removed from the Se-specific dataset due to overlap with genes altered by general toxicity and/or calorie restriction

GO CATEGORY - Biological Process	Total Genes[1]	Changed Genes[2]	Enrichment	FDR[3]
GO:0001957_intramembranous_ossification	5	3	16.47	0.015
GO:0006878_cellular_copper_ion_homeostasis	8	3	10.29	0.047
GO:0008354_germ_cell_migration	13	4	8.45	0.029
GO:0050819_negative_regulation_of_coagulation	24	6	6.86	0.008
GO:0046889_positive_regulation_of_lipid_biosynthetic_process	24	5	5.72	0.035
GO:0060349_bone_morphogenesis	24	5	5.72	0.035
GO:0050818_regulation_of_coagulation	36	7	5.34	0.009
GO:0033993_response_to_lipid	39	7	4.93	0.012
GO:0034329_cell_junction_assembly	41	7	4.69	0.021
GO:0031214_biomineral_formation	54	8	4.07	0.022
GO:0034330_cell_junction_organization	55	8	3.99	0.023
GO:0046890_regulation_of_lipid_biosynthetic_process	51	7	3.77	0.042
GO:0021537_telencephalon_development	62	8	3.54	0.039
GO:0031960_response_to_corticosteroid_stimulus	125	15	3.29	0.001
GO:0009612_response_to_mechanical_stimulus	75	9	3.29	0.039
GO:0048741_skeletal_muscle_fiber_development	76	9	3.25	0.037
GO:0048747_muscle_fiber_development	79	9	3.13	0.040
GO:0043627_response_to_estrogen_stimulus	143	16	3.07	0.004
GO:0048545_response_to_steroid_hormone_stimulus	272	30	3.03	0.000
GO:0051384_response_to_glucocorticoid_stimulus	118	13	3.02	0.012
GO:0007599_hemostasis	105	11	2.88	0.035
GO:0001666_response_to_hypoxia	174	18	2.84	0.004
GO:0031589_cell-substrate_adhesion	116	12	2.84	0.029
GO:0060348_bone_development	171	17	2.73	0.008
GO:0070482_response_to_oxygen_levels	185	18	2.67	0.008
GO:0001503_ossification	164	15	2.51	0.028
GO:0042060_wound_healing	208	19	2.51	0.009
GO:0050878_regulation_of_body_fluid_levels	146	13	2.44	0.047
GO:0007584_response_to_nutrient	193	17	2.42	0.021
GO:0001525_angiogenesis	182	16	2.41	0.029
GO:0016053_organic_acid_biosynthetic_process	161	14	2.39	0.041
GO:0046394_carboxylic_acid_biosynthetic_process	161	14	2.39	0.041

GO:0001568_blood_vessel_development	278	24	2.37	0.004
GO:0014706_striated_muscle_tissue_development	174	15	2.37	0.037
GO:0009725_response_to_hormone_stimulus	468	40	2.35	0.000
GO:0001944_vasculature_development	282	24	2.34	0.005
GO:0022603_regulation_of_anatomical_structure_morphogenesis	260	22	2.32	0.009
GO:0048514_blood_vessel_morphogenesis	237	20	2.32	0.012
GO:0006631_fatty_acid_metabolic_process	216	18	2.29	0.028
GO:0035295_tube_development	206	17	2.27	0.034
GO:0060537_muscle_tissue_development	182	15	2.26	0.048
GO:0009719_response_to_endogenous_stimulus	524	42	2.20	0.000
GO:0016477_cell_migration	375	30	2.20	0.001
GO:0042493_response_to_drug	301	24	2.19	0.009
GO:0032787_monocarboxylic_acid_metabolic_process	317	25	2.17	0.010
GO:0007517_muscle_organ_development	245	19	2.13	0.038
GO:0048870_cell_motility	403	31	2.11	0.004
GO:0031667_response_to_nutrient_levels	261	20	2.10	0.034
GO:0032989_cellular_component_morphogenesis	423	32	2.08	0.004
GO:0008610_lipid_biosynthetic_process	306	23	2.06	0.023
GO:0000902_cell_morphogenesis	386	29	2.06	0.008
GO:0009991_response_to_extracellular_stimulus	284	21	2.03	0.038
GO:0008284_positive_regulation_of_cell_proliferation	401	29	1.99	0.011
GO:0048646_anatomical_structure_formation_involved_in_morphogenesis	346	25	1.98	0.023
GO:0006928_cell_motion	548	39	1.95	0.001
GO:0051674_localization_of_cell	548	39	1.95	0.001
GO:0009888_tissue_development	662	47	1.95	0.000
GO:0044255_cellular_lipid_metabolic_process	606	43	1.95	0.001
GO:0009628_response_to_abiotic_stimulus	383	27	1.94	0.023
GO:0010033_response_to_organic_substance	855	59	1.89	0.000
GO:0019752_carboxylic_acid_metabolic_process	538	37	1.89	0.006
GO:0043436_oxoacid_metabolic_process	538	37	1.89	0.006
GO:0006082_organic_acid_metabolic_process	542	37	1.87	0.008
GO:0042180_cellular_ketone_metabolic_process	545	37	1.86	0.007
GO:0007010_cytoskeleton_organization	417	28	1.84	0.033
GO:0040011_locomotion	487	32	1.80	0.023
GO:0007155_cell_adhesion	586	38	1.78	0.011
GO:0022610_biological_adhesion	586	38	1.78	0.011
GO:0042127_regulation_of_cell_proliferation	742	48	1.78	0.004

GO:0006629_lipid_metabolic_process	695	44	1.74	0.009
GO:0009653_anatomical_structure_morphogenesis	1158	72	1.71	0.000
GO:0009605_response_to_external_stimulus	929	57	1.68	0.001
GO:0042221_response_to_chemical_stimulus	1379	83	1.65	0.000
GO:0032879_regulation_of_localization	608	36	1.63	0.047
GO:0009887_organ_morphogenesis	735	43	1.61	0.032
GO:0048869_cellular_developmental_process	1545	89	1.58	0.000
GO:0008283_cell_proliferation	1007	58	1.58	0.010
GO:0048513_organ_development	1633	92	1.55	0.000
GO:0030154_cell_differentiation	1480	83	1.54	0.001
GO:0048856_anatomical_structure_development	2224	121	1.49	0.000
GO:0048731_system_development	2107	113	1.47	0.000
GO:0050793_regulation_of_developmental_process	1181	63	1.46	0.030
GO:0048523_negative_regulation_of_cellular_process	1366	72	1.45	0.018
GO:0048519_negative_regulation_of_biological_process	1500	79	1.45	0.010
GO:0007275_multicellular_organismal_development	2439	125	1.41	0.000
GO:0006950_response_to_stress	1467	75	1.40	0.029
GO:0032502_developmental_process	2955	149	1.38	0.000
GO:0050896_response_to_stimulus	2589	121	1.28	0.023
GO:0032501_multicellular_organismal_process	3222	149	1.27	0.008
GO:0065007_biological_regulation	5146	214	1.14	0.038

[1] Number of genes involved in this process in the set of all genes on the Rat Genome 230 2.0 Array
[2] Number of genes involved in this process in the set of overlapping genes (genes in the original 1193-transcript dataset that overlapped with genes altered by general toxicity and/or calorie restriction)
[3] False Discovery Rate - ratio of the number of times the overlapping genes data gave P<0.05 compared to a random set of data
